# Artificial Intelligence and Employment: New Cross-Country Evidence

**DOI:** 10.3389/frai.2022.832736

**Published:** 2022-05-10

**Authors:** Alexandre Georgieff, Raphaela Hyee

**Affiliations:** Organisation for Economic Co-operation and Development, Paris, France

**Keywords:** J21, J23, J24, O33, artificial intelligence

## Abstract

Recent years have seen impressive advances in artificial intelligence (AI) and this has stoked renewed concern about the impact of technological progress on the labor market, including on worker displacement. This paper looks at the possible links between AI and employment in a cross-country context. It adapts the *AI occupational impact measure* developed by Felten, Raj and Seamans—an indicator measuring the degree to which occupations rely on abilities in which AI has made the most progress—and extends it to 23 OECD countries. Overall, there appears to be no clear relationship between AI exposure and employment growth. However, in occupations where computer use is high, greater exposure to AI is linked to higher employment growth. The paper also finds suggestive evidence of a negative relationship between AI exposure and growth in average hours worked among occupations where computer use is low. One possible explanation is that partial automation by AI increases productivity directly as well as by shifting the task composition of occupations toward higher value-added tasks. This increase in labor productivity and output counteracts the direct displacement effect of automation through AI for workers with good digital skills, who may find it easier to use AI effectively and shift to non-automatable, higher-value added tasks within their occupations. The opposite could be true for workers with poor digital skills, who may not be able to interact efficiently with AI and thus reap all potential benefits of the technology^1^.

## Introduction

Recent years have seen impressive advances in Artificial Intelligence (AI), particularly in the areas of image and speech recognition, natural language processing, translation, reading comprehension, computer programming, and predictive analytics.

This rapid progress has been accompanied by concern about the possible effects of AI deployment on the labor market, including on worker displacement. There are reasons to believe that its impact on employment may be different from previous waves of technological progress. Autor et al. (2003) postulated that jobs consist of routine (and thus in principle programmable) and non-routine tasks. Previous waves of technological progress were primarily associated with the automation of routine tasks. Computers, for example, are capable of performing routine cognitive tasks including record-keeping, calculation, and searching for information. Similarly, industrial robots are programmable manipulators of physical objects and therefore associated with the automation of routine manual tasks such as welding, painting or packaging (Raj and Seamans, 2019)^2^. These technologies therefore mainly substitute for workers in low- and middle-skill occupations.

Tasks typically associated with high-skilled occupations, such as non-routine manual tasks (requiring dexterity) and non-routine cognitive tasks (requiring abstract reasoning, creativity, and social intelligence) were previously thought to be outside the scope of automation (Autor et al., 2003; Acemoglu and Restrepo, 2020).

However, recent advances in AI mean that non-routine cognitive tasks can also increasingly be automated (Lane and Saint-Martin, 2021). In most of its current applications, AI refers to computer software that relies on highly sophisticated algorithmic techniques to find patterns in data and make predictions about the future. Analysis of patent texts suggests AI is capable of formulating medical prognosis and suggesting treatment, detecting cancer and identifying fraud (Webb, 2020). Thus, in contrast to previous waves of automation, AI might disproportionally affect high-skilled workers.

Even if AI automates non-routine, cognitive tasks, this does not necessarily mean that AI will displace workers. In general, technological progress improves labor efficiency by (partially) taking over/speeding up tasks performed by workers. This leads to an increase in output per effective labor input and a reduction in production costs. The employment effects of this process are ex-ante ambiguous: employment may fall as tasks are automated (substitution effect). On the other hand, lower production costs may increase output if there is sufficient demand for the good/service (productivity effect)^3^.

To harness this productivity effect, workers need to both learn to work effectively with the new technology and to adapt to a changing task composition that puts more emphasis on tasks that AI cannot yet perform. Such adaptation is costly and the cost will depend on worker characteristics.

The areas where AI is currently making the most progress are associated with non-routine, cognitive tasks often performed by medium- to high-skilled, white collar workers. However, these workers also rely more than other workers on abilities AI does not currently possess, such as inductive reasoning or social intelligence. Moreover, highly educated workers often find it easier to adapt to new technologies because they are more likely to already work with digital technologies and participate more in training, which puts them in a better position than lower-skilled workers to reap the potential benefits of AI. That being said, more educated workers also tend to have more task-specific human capital^4^, which might make adaption more costly for them (Fossen and Sorgner, 2019).

As AI is a relatively new technology, there is little empirical evidence on its effect on the labor market to date. The literature that does exist is mostly limited to the US and finds little evidence for AI-driven worker displacement (Lane and Saint-Martin, 2021). Felten et al. (2019) look at the effect of exposure to AI^5^ on employment and wages in the US at the occupational level. They do not find any link between AI exposure and (aggregate) employment, but they do find a positive effect of AI exposure on wage growth, suggesting that the productivity effect of AI may outweigh the substitution effect. This effect on wage growth is concentrated in occupations that require software skills and in high-wage occupations.

Again for the US, Fossen and Sorgner (2019) look at the effect of exposure to AI^6^ on job stability and wage growth at the individual level. They find that exposure to AI leads to higher employment stability and higher wages, and that this effect is stronger for higher educated and more experienced workers, again indicating that the productivity effect dominates and that it is stronger for high-skilled workers.

Finally, Acemoglu et al. (2020) look at hiring in US firms with task structures compatible with AI capabilities^7^. They find that firms' exposure to AI is linked to changes in the structure of skills that firms demand. They find no evidence of employment effects at the occupational level, but they do find that firms that are exposed to AI restrict their hiring in non-AI positions compared to other firms. They conclude that the employment effect of AI might still be too small to be detected in aggregate data (given also how recent a phenomenon AI is), but that it might emerge in the future as AI adoption spreads.

This paper adds to the literature by looking at the links between AI and employment growth in a cross-country context. It adapts the *AI occupational impact measure* proposed by Felten et al. (2018, 2019)—an indicator measuring the degree to which occupations rely on abilities in which AI has made the most progress in recent years—and extends it to 23 OECD countries by linking it to the Survey of Adult Skills, PIAAC. This indicator, which allows for variations in AI exposure across occupations, as well as within occupations and across countries, is matched to Labor Force Surveys to analyse the relationship with employment growth.

The paper finds that, over the period 2012–2019, there is no clear relationship between AI exposure and employment growth across all occupations. Moreover, in occupations where computer use is high, AI appears to be positively associated with employment growth. There is also some evidence of a negative relationship between AI exposure and growth in average hours worked among occupations where computer use is low. While further research is needed to identify the exact mechanisms driving these results, one possible explanation is that partial automation by AI increases productivity directly as well as by shifting the task composition of occupations toward higher value-added tasks. This increase in labor productivity and output counteracts the direct displacement effect of automation through AI for workers with good digital skills, who may find it easier to use AI effectively and shift to non-automatable, higher-value tasks within their occupations. The opposite could be true for workers with poor digital skills, who may be unable to interact efficiently with AI and thus reap all potential benefits of the technology.

The paper starts out by presenting indicators of AI deployment that have been proposed in the literature and discussing their relative merits (Section Indicators of Occupational Exposure to AI). It then goes on to present the indicator developed in this paper and builds some intuition on the channels through which occupations are potentially affected by AI (Section Data). Section Results presents the main results.

## Indicators of Occupational Exposure to AI

To analyse the links between AI and employment, it is necessary to determine where in the economy AI is currently deployed. In the absence of comprehensive data on the adoption of AI by firms, several proxies for (potential) AI deployment have been proposed in the literature. They can be grouped into two broad categories. The first group of indicators uses information on labor demand to infer AI activity across occupations, sectors and locations. In practice, these indicators use online job postings that provide information on skills requirements and they therefore will only capture AI deployment if it requires workers to have AI skills. The second group of indicators uses information on AI capabilities—that is, information on what AI can currently do—and links it to occupations. These indicators measure potential exposure to AI and not actual AI adoption. This section presents some of these indicators and discusses their advantages and drawbacks.

### Indicators Based on AI-Related Job Posting Frequencies

The first set of indicators use data on AI-related skill requirements in job postings as a proxy for AI deployment in firms. The main data source for these indicators is Burning Glass Technologies (BGT), which collects detailed information—including job title, sector, required skills etc. —on online job postings (see Box 1 for details). Because of the rich and up-to-date information BGT data offers, these indicators allow for a timely tracking of the demand for AI skills across the labor market.

Box 1 **Burning Glass Technologies (BGT) online job postings data**

Burning Glass Technologies (BGT) collects data on online job postings by web-scraping 40 000 online job boards and company websites. It claims to cover the near-universe of online job postings. Data are currently available for Australia, Canada, New Zealand, Singapore, the United Kingdom, and the United States for the time period 2012–2020 (2014–2020 for Germany and 2018–2020 for other European Union countries). BGT extracts information such as location, sector, occupation, required skills, education, and experience levels from the text of job postings (deleting duplicates) and organizes it into up to 70 variables that can be linked to labor force surveys, providing detailed, and timely information on labor demand.

Despite its strengths, BGT data has a number of limitations:

- It misses vacancies that are not posted online. Carnevale et al. (2014) compare vacancies from survey data according to the Job Openings and Labor Turnover Survey (JOLTS) from the US Bureau of Labor Statistics, a representative survey of 16,000 US businesses, with BGT data for 2013. They find that roughly 70% of vacancies were posted online, with vacancies requiring a college degree significantly more likely to be posted online compared to jobs with lower education requirements.
  - There is not necessarily a direct, one-to-one correspondence between an online job ad and an actual vacancy: firms might post one job ad for several vacancies, or post job ads without firm plans to hire, e.g., because they want to learn about available talent for future hiring needs.
- BGT data might over-represent growing firms that cannot draw on internal labor markets to the same extent as the average firm.
- Higher turnover in some occupations and industries can produce a skewed image of actual labor demand since vacancies reflect a mixture of replacement demand as well as expansion.

In addition, since BGT data draws on published job advertisements, it is a proxy of current vacancies, and not of hiring or actual employment. As a proxy for vacancies, BGT data performs reasonably well, although some occupations and sectors are over-represented. Hershbein and Kahn (2018) show for the US that, compared to vacancy data from the U.S. Bureau of Labor Statistics' Job Openings and Labor Turnover Survey (JOLTS), BGT over-represents health care and social assistance, finance and insurance, and education, while under-representing accommodation, food services and construction (where informal hiring is more prevalent) as well as public administration/government. These differences are stable across time, however, such that *changes* in labor demand in BGT track well with JOLTS data. Regarding hiring, they also compare BGT data with new jobs according to the Current Population Survey (CPS). BGT data strongly over-represents computer and mathematical occupations (by a factor of over four, which is a concern when looking at growth in demand for AI skills as compared to other skills), as well as occupations in management, healthcare, and business and financial operations. It under-represents all remaining occupations, including transportation, food preparation and serving, production, or construction.

Cammeraat and Squicciarini (2020) argue that, because of differences in turnover across occupations, countries and time, as well as differences in the collection of national vacancy statistics, the representativeness of BGT data as an indicator for labor and skills demand should be measured against employment growth. They compare growth rates in employment with growth rates in BGT job postings on the occupational level in the six countries for which a BGT timeline exists. They find that, across countries, the deviation between BGT and employment growth rates by occupation is lower than 10 percentage points for 65% of the employed population. They observe the biggest deviations for agricultural, forestry and fishery workers, as well as community and personal service workers, again occupations where informal hiring may be more prevalent.

Squicciarini and Nachtigall (2021) identify AI-related job postings by using keywords extracted from scientific publications, augmented by text mining techniques and expert validation [see Baruffaldi et al. (2020) for details]. These keywords belong to four broad groups: (i) generic AI keywords, e.g., “artificial intelligence,” “machine learning;” (ii) AI approaches or methods: e.g., “decision trees,” “deep learning;” (iii) AI applications: e.g., “computer vision,” “image recognition;” (iv) AI software and libraries: e.g., Python or TensorFlow. Since some of these keywords may be used in job postings for non AI-related jobs (e.g., “Python” or “Bayesian”), the authors only tag a job as AI-related if the posting contains at least two AI keywords from at least two distinct concepts. This indicator is available on an annual basis for Canada, Singapore, the United Kingdom and the United States, for 2012–2018^8^.

Acemoglu et al. (2020) take a simpler approach by defining vacancies as AI-related if they contain any keyword belonging to a simple list of skills related to AI^9^. As this indicator will tag any job posting that contains one of the keywords, it is less precise than the indicator proposed by Squicciarini and Nachtigall (2021), but also easier to reproduce.

Dawson et al. (2021) develop the *skills-space* or *skills-similarity* indicator. This approach defines two skills as similar if they often occur together in BGT job postings and are both simultaneously important for the job posting. A skill is assumed to be less “important” for a particular job posting if it is common across job postings. For example, “communication” and “team work” occur in about a quarter of all job adds, and would therefore be less important than “machine learning” in a job posting requiring both “communication” and “team work^10^.” The idea behind this approach is that, if two skills are often simultaneously required for jobs, (i) they are complementary and (ii) mastery of one skill means it is easier to acquire the other. In that way, similar skills may act as “bridges” for workers wanting to change occupations. It also means that workers who possess skills that are similar to AI skills may find it easier to work with AI, even if they are not capable of developing the technology themselves. For example, the skill “copy writing” is similar to “journalism,” meaning that a copy writer might transition to journalism at a lower cost than, say, a social worker, and that a copy writer might find it comparatively easier to use databases and other digital tools created for journalists.

Skill similarity allows the identification and tracking of emerging skills: using a short list of “seed skills^11^,” the indicator can track similar skills as they appear in job ads over time, keeping the indicator up to date. For example, TensorFlow is a deep learning framework introduced in 2016. Many job postings now list it as a requirement without additionally specifying “deep learning” (Dawson et al., 2021).

The skill similarity approach is preferable to the simple job posting frequency indicators mentioned above (Acemoglu et al., 2020; Squicciarini and Nachtigall, 2021) as it does not only pick up specific AI job postings, but also job postings with skills that are similar (but not identical) to AI skills, and may thus enable workers to work with AI technologies. Another advantage of this indicator is its dynamic nature: as technologies develop and skill requirements evolve, skill similarity can identify new skills that appear in job postings together with familiar skills, and keep the relative skill indicators up-to-date. This indicator is available at the annual level from 2012 to 2019 for Australia and New Zealand^12^.

### Task-Based Indicators

Task-based indicators for AI adoption are based on measures of AI capabilities linked to tasks workers perform, often at the occupational level. They identify occupations as exposed to AI if they perform tasks that AI is increasingly capable of performing.

The *AI occupational exposure measure* developed by Felten et al. (2018, 2019) is based on progress scores in nine AI applications^13^ (such as reading comprehension or image recognition) from the AI progress measurement dataset provided by the Electronic Frontier Foundation (EFF). The EFF monitors progress in AI applications using a mixture of academic literature, blog posts and websites focused on AI. Each application may have several progress scores. One example of a progress score would be a recognition error rate for image recognition. The authors rescale these scores to arrive at a composite score that measures progress in each application between 2010 and 2015.

Felten et al. (2018, 2019) then link these AI applications to abilities in the US Department of Labor's O^*^NET database. Abilities are defined as “enduring attributes of the individual that influence performance,” e.g., “peripheral vision” or “oral comprehension.” They enable workers to perform tasks in their jobs (such as driving a car or answering a call), but are distinct from skills in that they cannot typically be acquired or learned. Thus, linking O^*^NET abilities to AI applications means linking human to AI abilities.

The link between O^*^NET abilities and AI applications (a correlation matrix) is made via an Amazon Mechanical Turk survey of 200 gig workers per AI application, who are asked whether a given AI application—e.g., image recognition—can be used for a certain ability—e.g., peripheral vision^14^. The correlation matrix between applications and abilities is then calculated as the share of respondents who thought that a given AI application could be used for a given ability. These abilities are subsequently linked to occupations using the O^*^NET database. This indicator is available for the US for 2010–2015^15^.

Similarly, the *Suitability for Machine Learning* indicator developed by Brynjolfsson and Mitchell (2017); Brynjolfsson et al. (2018) assigns a suitability for machine learning score to each of the 2,069 narrowly defined work activities from the O^*^NET database that are shared across occupations (e.g., “assisting and caring for others,” “coaching others,” “coordinating the work of others”). For these scores, they use a Machine Learning suitability rubric consisting of 23 distinct statements describing a work activity. For example, for the statement “Task is describable by rules,” the highest score would be “Task can be fully described by a detailed set of rules (e.g., following a recipe),” whereas the lowest score would be “The task has no clear, well-known set of rules on what is and is not effective (e.g., writing a book).” They use the human intelligence task crowdsourcing platform CrowdFlower to score each direct work activity by seven to ten respondents. The direct work activities are then aggregated to tasks (e.g., “assisting and caring for others,” “coaching others,” “coordinating the work of others” aggregate to “interacting with others”), and the tasks to occupations. This indicator is available for the US for the year 2016/2017.

Tolan et al. (2021) introduce a layer of cognitive abilities to connect AI applications (that they call benchmarks) to tasks. The authors define 14 cognitive abilities (e.g., visual processing, planning and sequential decision-making and acting, communication, etc.) from the psychometrics, comparative psychology, cognitive science, and AI literature^16^. They link these abilities to 328 different AI benchmarks (or applications) stemming from the authors' own previous analysis and annotation of AI papers as well as from open resources such as Papers with Code^17^. These sources in turn draw on data from multiple verified sources, including academic literature, review articles etc. on machine learning and AI. They use the research intensity in a specific benchmark (number of publications, news stories, blog entries etc.) obtained from AI topics^18^. Tasks are measured at the worker level using the European Working Conditions Survey (EWCS), PIAAC and the O^*^NET database. Task intensity is derived as a measure of how much time an individual worker spends on a task and how often the task is performed.

The mapping between cognitive abilities and AI benchmarks, as well as between cognitive abilities and tasks, relies on a correspondence matrix that assigns a value of 1 if the ability is absolutely required to solve a benchmark or complete a task, and 0 if it is not necessary at all. This correspondence matrix was populated by a group of multidisciplinary researchers for the mapping between tasks and cognitive abilities, and by a group of AI-specialized researchers for the mapping between AI benchmarks and cognitive abilities. This indicator is available from 2008 to 2018, at the ISCO-3 level, and constructed to be country-invariant (as it combines data covering different countries).

Webb (2020) constructs his *exposure of occupations to any technology* indicator by directly comparing the text of patents from Google patents public data to the texts of job descriptions from the O^*^NET database to quantify the overlap between patent descriptions and job task descriptions. By limiting the patents to AI patents (using a list of key-words), this indicator can be narrowed to only apply to AI. Each particular task is then assigned a score according to the prevalence of such patents that mention this task; tasks are then aggregated to occupations.

### What Do These Indicators Measure?

To gauge the link between AI and employment, the chosen indicator for this study should proxy actual AI deployment in the economy as closely as possible. Furthermore, it should proxy AI deployment at the occupation level because switching occupations is more costly for workers than switching firms or sectors, making the occupation the relevant level for the automation risk of individual workers.

Task-based approaches measure *potential automatability* of tasks (and occupations), so they are measures of AI exposure, not deployment. Because task-based measures look at potential automatability, they cannot capture uneven adoption of AI across occupations, sectors or countries. Thus, in a cross-country analysis, the only source of variation in a task-based indicator are differences in the occupational task composition across countries, as well as cross-country differences in the occupational distribution.

Indicators based on job posting data measure *demand for AI skills* (albeit with some noise, see Box 1), as opposed to *AI use*. Thus, they rely on the assumption that AI use in a firm, sector or occupation will lead to employer demand for AI skills *in that particular firm, sector, or occupation*. This is not necessarily the case, however:

- Some firms will decide to train workers in AI rather than recruit workers with AI skills; their propensity to do so may vary across occupations.
- Many AI applications will not require AI skills to work with them.
- Even where AI skills are needed, many firms, especially smaller ones, are likely to outsource AI development and support with its adoption to specialized AI development firms. In this case, vacancies associated with AI adoption would emerge in a different firm or sector to where the technology was actually being deployed.
- The assumption that AI deployment requires hiring of staff with AI skills is even more problematic when the indicator is applied at the occupation level. Firms that adopt AI may seek workers with AI skills in completely different occupations than the workers whose tasks are being automated by AI. For instance, an insurance company wanting to substitute or enhance some of the tasks of insurance clerks with AI would not necessarily hire insurance clerks with AI skills, but AI professionals to develop or deploy the technology. Insurance clerks may only have to interact with this technology, which might not require AI development skills (but may well-require other specialized skills). Thus, even with broad-based deployment of AI in the financial industry, this indicator may not show an increasing number of job postings for insurance clerks with AI skills. This effect could also be heterogeneous across countries and time. For example, Qian et al. (2020) show that law firms in the UK tend to hire AI professionals without legal knowledge, while law firms in Singapore and the US do advertise jobs with hybrid legal-AI skillsets.

Thus, indicators based on labor demand data are a good proxy for AI deployment at the firm and sector level as long as there is no significant outsourcing of AI development and maintenance, and the production process is such that using the technology requires specialized AI skills. If these assumptions do not hold, these indicators will be incomplete. Whether or not this is the case is an empirical question that requires further research. To date the only empirical reference on this question is Acemoglu et al. (2020) who show for the US that the share of job postings that require AI skills increases faster in firms that are heavily exposed to AI (according to task-based indicators). For example, a one standard deviation increase in the measure of AI exposure according to Felten et al. (2018, 2019) leads to a 15% increase in the number of published AI vacancies.

**Figure 1:**
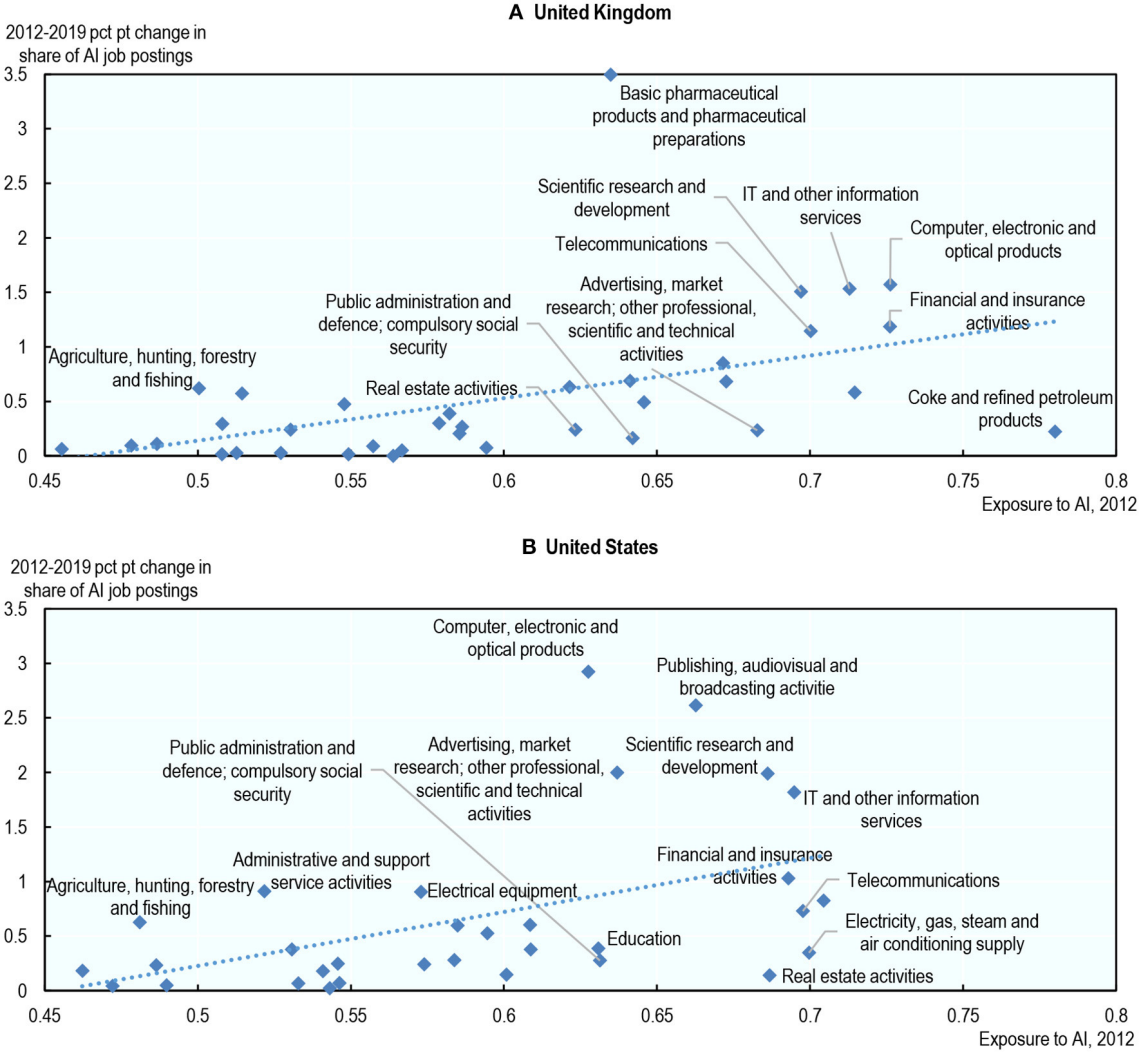
Sectors with higher exposure to AI saw a higher increase in their share of job postings that require AI skills. Percentage point* change in the share of job postings that require AI skills (2012–2019) vs. average exposure to AI (2012), by sector. The share of job postings that require AI skills in a sector is the number of job postings requiring such skills in that sector divided by the total number of job postings in that same sector. Not all sectors have marker labels due to space constraints. *Percentage point changes are preferred over percentage changes because the share of job postings that require AI skills is equal to zero in some sectors in 2012. Source: Author' calculations using data from Burning Glass Technologies, PIAAC and Felten et al. (2019). **(A)** United Kingdom and **(B)** United States.

To shed further light on the relationship between the two types of indicators, Figure 1 plots the 2012–2019 percentage point change in the share of BGT job postings that require AI skills^19^ across 36 sectors against a sector-level task-based AI exposure score, similar to the occupational AI exposure score developed in this paper (see Section Construction of the AI Occupational Exposure Measure)^20^. This analysis only covers the United Kingdom and the United States^21^ because of data availability. For both countries, a positive relationship is apparent, suggesting that, overall, (i) the two measures are consistent and (ii) AI deployment does require some AI talent *at the sector level*. Specifically, a one standard deviation increase in AI exposure (approximately the difference in exposure between finance and public administration) is associated with a 0.33 higher percentage point change in the share of job postings that require AI skills in the United-Kingdom; a similar relationship emerges in the United-States^22^.

While it is reassuring that, at the sector level, the two measures appear consistent, it is also clear that job postings that require AI skills fail to identify certain sectors that are, from a task perspective, highly exposed to AI, such as education, the energy sector, the oil industry, public administration and real estate activities. This suggests that AI development and support may be outsourced and/or that the use of AI does not require AI skills in these sectors.

In addition, and as stated above, there is a priori no reason that demand-based indicators would pick up AI deployment at the occupational level, as firms that adopt AI may seek workers with AI skills in completely different occupations than the workers whose tasks are being automated by AI. This is also borne out in the analysis in this paper (see Section Exposure to AI and Demand for AI-Related Technical Skills: A Weak but Positive Relationship Among Occupations Where Computer Use is High). Thus, labor demand-based indicators are unlikely to be good proxies for AI deployment at the occupational level and, in the analysis described in this paper, preference will be given to task-based measures even though they, too, are only an imperfect proxy for AI adoption.

### Which Employment Effects Can These Indicators Capture?

This paper analyses the relationship between AI adoption and employment at the occupational level, since it is automation risk at the occupational level that is most relevant for individual workers. The analysis will therefore require a measure of AI adoption at the occupational level and this section assesses which type of indicator might be best suited to that purpose.

It is useful to think of AI-driven automation as having two possible, but opposed, employment effects. On the one hand, AI may depress employment via automation/substitution. On the other, it may increase it by raising worker productivity.

Focusing on the substitution effect first, task-based indicators will pick up such effects since they measure what tasks could potentially be automated by AI. By contrast, labor-demand based indicators identify occupational AI exposure only if AI skills are mentioned in online job postings for a particular occupation. Thus, they will only pick up substitution effects (that is, a subsequent decline in employment for a particular occupation) if the production process is such that workers whose tasks are being automated need AI skills to interact with the technology.

Regarding the productivity effect, there are several ways in which AI might increase employment. The most straightforward way is that AI increases productivity in a given task, and thus lowers production costs, which can lead to increased employment if demand for a product or service is sufficiently price elastic. This was the case, for example, for weavers in the industrial revolution [see Footnote 4, Bessen (2016)].

In addition, technological progress may allow workers to focus on higher value-added tasks within their occupation that the technology cannot (yet) perform. For example, AI is increasingly deployed in the financial services industry to forecast stock performance. Grennan and Michaely (2017) show that stock analysts have shifted their attention away from stocks for which an abundance of data is available (which lends itself to analysis by AI) toward stocks for which data is scarce. To predict the performance of “low-AI” stocks, analysts gather “soft” information directly from companies' management, suppliers and clients, thus concentrating on tasks requiring a capacity for complex human interaction, of which AI is not (yet) capable.

Task-based indicators will pick up these productivity effects (as they identify exposed occupations directly via their task structure), while labor-demand based indicators will only do so if workers whose tasks are being automated need to interact with the technology, and interacting with the technology requires specialized AI skills.

AI can also be used to augment other technologies, that subsequently automate certain tasks. For example, in robotics, AI supports the efficient automation of physical tasks by improving the vision of robots, or by enabling robots to “learn” from the experience of other robots, e.g., by facilitating the exchange of information on the layout of rooms between cleaning robots (Nolan, 2021). While these improvements to robotics are connected to AI applications (in this example: image recognition and sensory perception of room layouts), the tasks that are being automated (cleaning of rooms) mostly consist of the physical manipulation of objects and thus pertain to the field of robotics. Thus, AI improves the effectiveness of robots to perform tasks associated with cleaners, without performing physical cleaning tasks. As task-based indicators only identify tasks that AI itself can perform (and not tasks that it merely facilitates), they would not capture this effect. In robotics, this would mostly affect physical tasks often performed by low and medium-skilled workers. Indicators based on online vacancies would also be unlikely to capture AI augmenting other technologies at the occupation level—unless cleaners require AI skills to work with cleaning robots.

Finally, AI could enable the launch of completely new products or services, that lead to job creation, e.g., in marketing or sales of AI-based products and services (Acemoglu et al., 2020). Both task- and labor-demand-based indicators cannot generally measure this effect (unless marketing/selling of AI products requires AI-skills).

To conclude, both types of indicators are likely to understate actual AI deployment at the occupational level (see Table 1). Labor-demand based indicators in particular will miss a significant part of AI deployment if workers whose tasks are being automated do not need to interact with AI or if the use of AI does not require any AI skills. Task-based indicators, on the other hand, are not capable of picking up differences in actual AI deployment across time and space (this is because they only measure exposure, not actual adoption). Finally, neither indicator will capture AI augmenting other automating technologies, such as robotics, which is likely to disproportionally affect low-skilled, blue collar occupations.

**Table 1:** Which potential employment effects of AI can task-based and labor-demand based indicators capture?

	**Task-Based indicators**	**Labor demand-based indicators**
Substitution effect (–)	Yes	Only if the production process is such that workers in the partially automated occupation require AI skills to interact with the technology
Productivity effect (+)	Yes	Only if the production process is such that workers in the partially automated occupation require AI skills to interact with the technology
Augmentation of other technologies (e.g., robotics) (–)	No	Only if the production process is such that workers in the partially automated occupation require AI skills to interact with the technology
Job creation through new products and services enabled by AI (+)	No	Only if these new jobs require AI skills

*The table only refers to employment effects identified at the occupational level. +/– denote the sign of the employment effect*.

On the whole, for assessing the links between AI and employment at the occupational level, indicators based on labor demand data are likely to be incomplete. Task-based indicators are therefore more appropriate for the analysis carried out in this paper. Keeping their limitations in mind, however, is crucial.

## Data

This paper extends the *occupational exposure measure*, proposed by Felten et al. (2018, 2019) to 23 OECD countries^23^ to look at the links between AI and labor market outcomes for 36 occupations^24^,^25^ in recent years (2012–2019). The *measure of occupational exposure to AI* proxies the degree to which tasks in those occupations can be automated by AI. Thus, the analysis compares occupations with a high degree of automatability by AI to those with a low degree.

This section presents the data used for the analysis. It begins by describing the construction of the measure of occupational exposure to AI developed and used in this paper, and builds some intuition as to why some occupations are exposed to a higher degree of potential automation by AI than others. It then shows some descriptive statistics for AI exposure and labor market outcomes: employment, working hours, and job postings that require AI skills. Finally, it describes different measures of the task composition of occupations, which will help shed light on the relationship between AI exposure and labor market outcomes.

### Occupational Exposure to AI

Several indicators for (potential) AI deployment have been proposed in the literature (see Section Indicators of Occupational Exposure to AI), most of them geared to the US. Since this paper looks at the links between AI and employment across several countries, country coverage is a key criterion for the choice of indicator. This excludes indicators based on AI-related job-posting frequencies, as pre-2018 BGT data is only available for English-speaking countries)^26^. In addition to data availability issues, indicators based on labor demand data are also likely to be less complete than task-based indicators (see Section What Do These Indicators Measure?). Among the task-based measures, the *suitability for machine learning* indicator (Brynjolfsson and Mitchell, 2017; Brynjolfsson et al., 2018) was not publicly accessible at the time of publication. Webb's (2020) indicator captures the stock of patents until 2020, and is therefore too recent to look at the links between AI and the labor market during the observation period (2012–2019), particularly given that major advancements in AI occurred between 2015 and 2020, and the slow pace of diffusion of technology in the economy. The paper therefore uses the occupational exposure measure (Felten et al., 2018, 2019), which has the advantage of capturing AI developments until 2015, leaving some time for the technology to be deployed in the economy. It is also based on actual scientific progress in AI, as opposed to research activity as the indicator proposed by Tolan et al. (2021).

While the preferred measure for this analysis is the *AI occupational exposure measure* proposed by Felten et al. (2018, 2019), the paper also presents additional results using Agrawal's, Gans and Goldfarb (2019) job-posting indicator (an indicator based on job postings), as well as robustness checks using task-based indicators by Webb (2020) and Tolan et al. (2021)^27^. This section describes the construction of the main indicator, and some descriptive statistics.

#### Construction of the AI Occupational Exposure Measure

**Figure 2:**
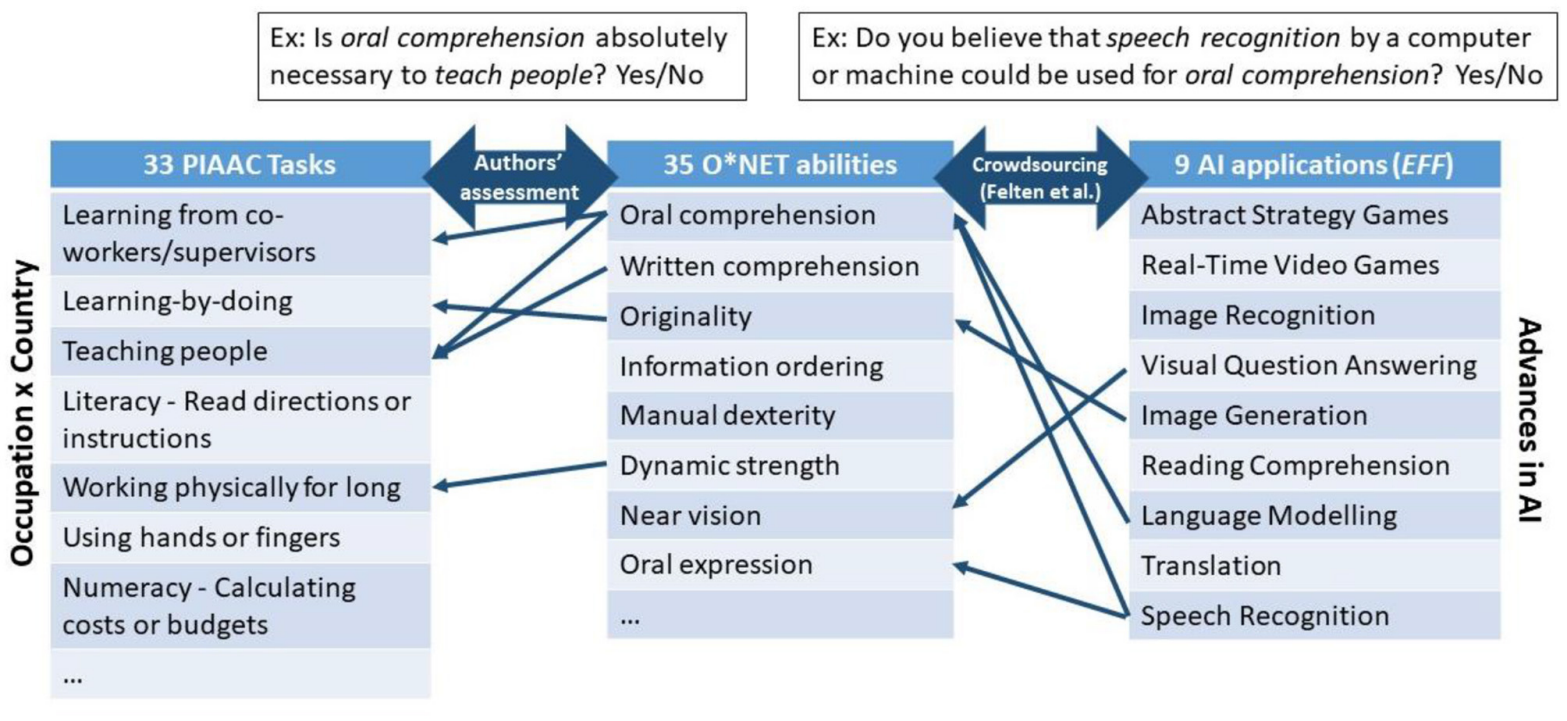
Construction of the measure of occupational exposure to AI. Adaptation from Felten et al. (2018) to 23 OECD countries. The authors link O*NET abilities and PIAAC tasks manually by asking whether a given ability is indispensable for performing a given task. The link between O*NET abilities and AI applications (a correlation matrix) is taken from Felten et al. (2019). The matrix was built by an Amazon Mechanical Turk survey of 200 gig workers per AI application, who were asked whether a given AI application can be used for a certain ability. The correlation matrix between applications and abilities is then calculated as the share of respondents who thought that a given AI application could be used for a given ability. This chart is for illustrative purposes and is not an exhaustive representation of the links between the tasks, abilities and AI applications displayed.

The *AI occupational exposure measure* links progress in nine AI applications to 52 abilities in the US Department of Labor's O^*^NET database (see Section What Do These Indicators Measure? for more details). This paper extends it to 23 OECD countries by mapping the O^*^NET abilities to tasks from the OECD's Survey of Adult Skills (PIAAC), and then back to occupations (see Figure 2 for an illustration of the link). Specifically, instead of using the O^*^NET US-specific measures of an ability's “prevalence” and “importance” in an occupation, country-specific measures have been developed based on data from PIAAC, which reports the frequency with which a number of tasks are performed on the job by each surveyed individual. This information was used to measure the average frequency with which workers in each occupation (classified using two-digit ISCO-08) perform 33 tasks, and this was done separately for each country. Each O^*^NET ability was then linked to each of these 33 tasks, based on the authors' binary assessments of whether the ability is needed to perform the task or not^28^.

This allows for task-content variations in AI exposure across occupations, as well as within occupations and across countries that may arise because of institutional or socio-economic differences across countries. Thus, the indicator proposed in this paper differs from that of Felten et al. (2019) only in that it relies on PIAAC data to take into account occupational task-content heterogeneity across countries. That is, the indicator adopted in this paper is defined at the occupation-country cell level rather than at the occupation level [as in Felten et al. (2019)]. It is scaled such that the minimum is zero and the maximum is one over the full sample of occupation-country cells. It indicates *relative exposure to AI*, and no other meaningful interpretation can be given to its actual values.

In this paper, the link between O^*^NET abilities and PIAAC tasks is performed manually by asking whether a given ability is indispensable for performing a given task, e.g., *is oral comprehension absolutely necessary to teach people*? A given O^*^NET ability can therefore be linked to several PIAAC tasks, and conversely, a given PIAAC task can be linked to several O^*^NET abilities. This link was made by the authors of the paper and, in case of diverging answers, agreement was reached through an iterative discussion and consensus method, similar to the Delphi method described in Tolan et al. (2021). Of the 52 O^*^NET abilities, 35 are related to at least one task in PIAAC. Thus, the indicator loses 17 abilities compared to Felten's et al. (2018, 2019) measure. All the measures that are lost in this way are physical, psychomotor or sensory, as there are no tasks requiring these abilities in PIAAC^29^. As a result, the occupational intensity of physical, psychomotor, or sensory abilities is poorly estimated using PIAAC data. Therefore, whenever possible, robustness checks use O^*^NET scores of “prevalence” and “importance” of abilities within occupations for the United States (as in Felten et al., 2018) instead of PIAAC-based measures. These robustness tests necessarily assume that the importance and prevalence of abilities are the same in other countries as in the United States. Another approach would have been to assign the EFF applications directly to the PIAAC tasks. However, we preferred to preserve the robustly established mapping of Felten et al. (2018).

The level of exposure to AI in a particular occupation reflects: (i) the progress made by AI in specific applications and (ii) the extent to which those applications are related to abilities required in that occupation. Like all task-based measures, it is at its core a measure of potential automation of occupations by AI, as it indicates which occupations rely most on abilities in which AI has made progress in recent years. It should capture potential positive productivity effects of AI, as well as negative substitution effects caused by (partial) automation of tasks by AI. However, it cannot capture any effects of AI progress on occupations when these effects do not rely on worker abilities that are directly related to the capabilities of AI, such as might be the case when AI augments other technologies, which consequently make progress in the abilities that a person needs in his/her job (see also Section What Do These Indicators Measure?). Section Occupational Exposure to AI shows AI exposure across occupations and builds some intuition on why the indicator identifies some occupations as more exposed to AI than others.

#### AI Progress and Abilities

**Figure 3:**
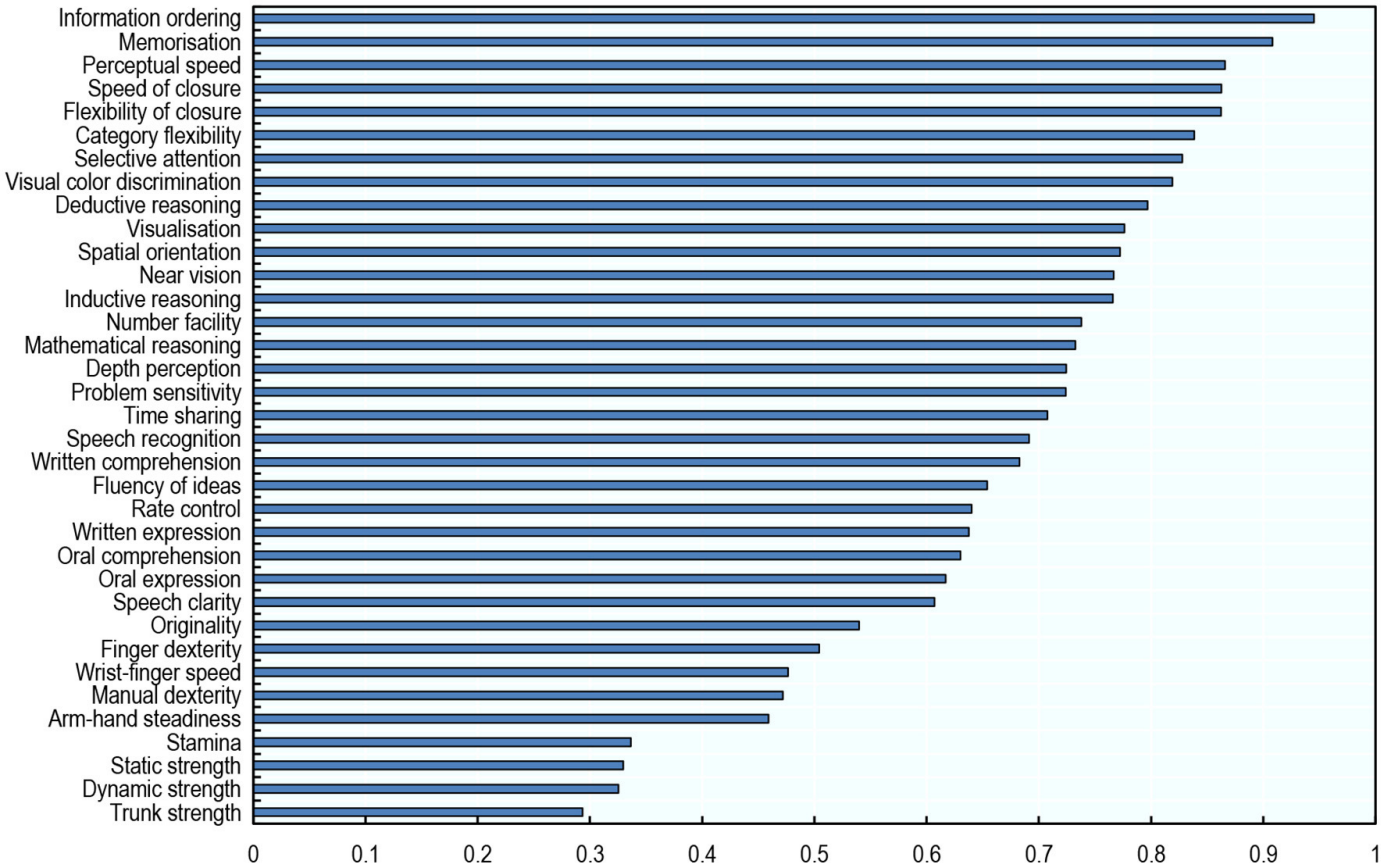
AI has made the most progress in abilities that are required to perform non-routine, cognitive tasks. Progress made by AI in relation to each ability, 2010–2015. The link between O*NET abilities and AI applications (a correlation matrix) is taken from Felten et al. (2019). The matrix was built by an Amazon Mechanical Turk survey of 200 gig workers per AI application, who were asked whether a given AI application—e.g., image recognition—can be used for a certain ability—e.g., near vision. The correlation matrix between applications and abilities is then calculated as the share of respondents who thought that a given AI application could be used for a given ability. To obtain the score of progress made by AI in relation to a given ability, the shares corresponding to that ability are first multiplied by the Electronic Frontier Foundation (EFF) progress scores in the AI applications; these products are then summed over all nine AI applications. Authors' calculations using data from Felten et al. (2019).

Over the period 2010–2015, AI has made the most progress in applications that affect abilities required to perform non-routine cognitive tasks, in particular: information ordering, memorisation, perceptual speed, speed of closure, and flexibility of closure (Figure 3)^30^. By contrast, AI has made the least progress in applications that affect physical and psychomotor abilities^31^. This is consistent with emerging evidence that AI is capable of performing cognitive, non-routine tasks (Lane and Saint-Martin, 2021).

#### Occupational Exposure to AI

**Figure 4:**
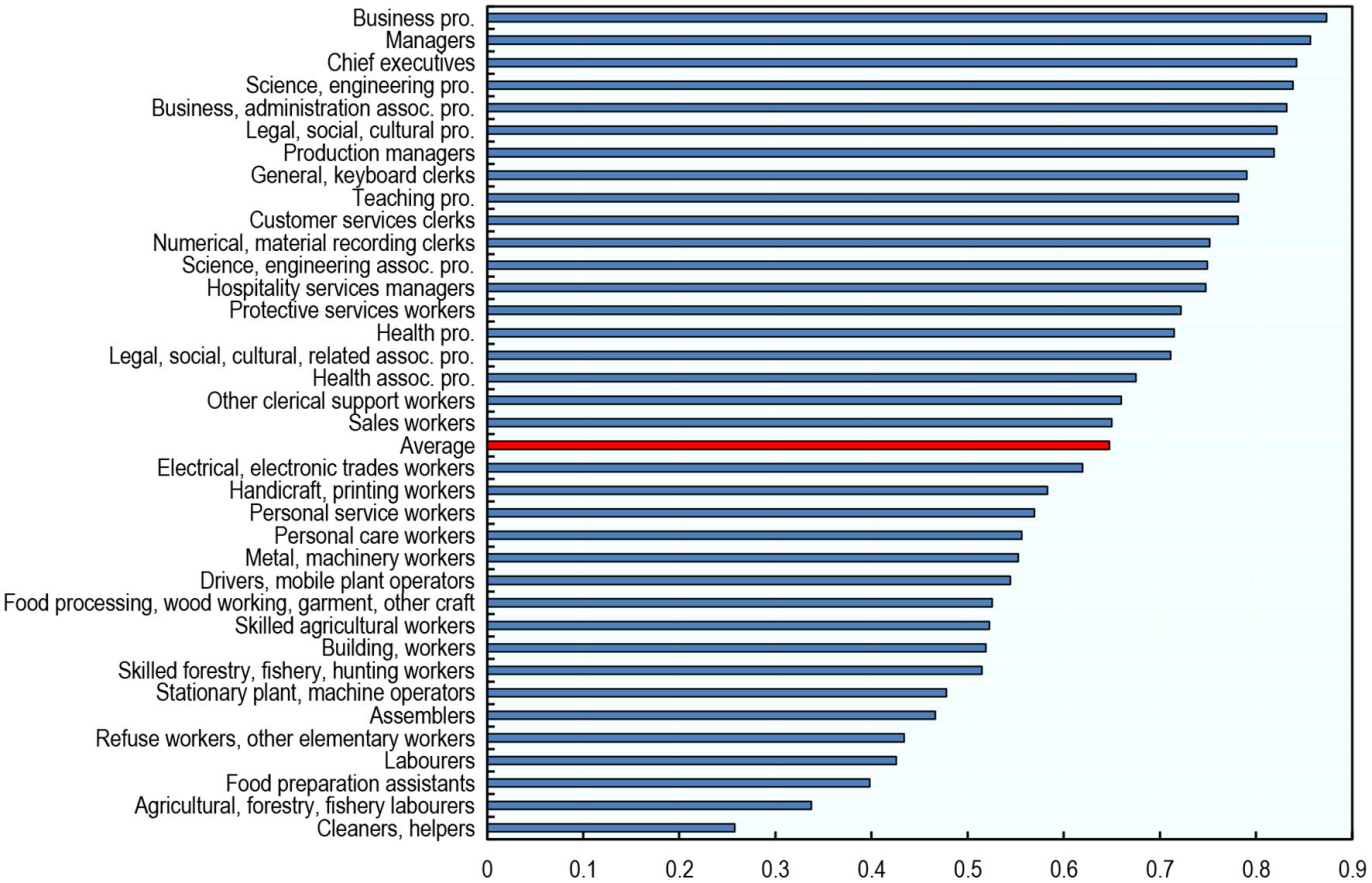
Highly educated white-collar occupations are among the occupations with the highest exposure to AI. Average exposure to AI across countries by occupation, 2012. The averages presented are unweighted. Cross-country averages are taken over the 23 countries included in the analysis. Authors' calculations using data from the Programme for the International Assessment of Adult Competencies (PIAAC) and Felten et al. (2019).

The kind of abilities AI has made the most progress in are disproportionately used in highly-educated, white-collar occupations. As a result, white-collar occupations requiring high levels of formal education are among the occupations with the highest exposure to AI: Science and Engineering Professionals, but also Business and Administration Professionals, Managers; Chiefs Executives; and Legal, Social, and Cultural Professionals (Figure 4). By contrast, occupations with the lowest exposure include occupations with an emphasis on physical tasks: Cleaners and Helpers; Agricultural Forestry, Fishery Laborers; Food Preparation Assistants and Laborers^32^.

The occupational intensity of some abilities is poorly estimated due to PIAAC data limitations. In particular, the 33 PIAAC tasks used in the analysis include only two non-cognitive tasks, and some of the O^*^NET abilities are not related to any of these tasks. Therefore, as a robustness exercise, Figure A A.1 displays the level of exposure to AI obtained when using O^*^NET scores of “prevalence” and “importance” of abilities within occupations for the United States (as in Felten et al., 2018) instead of the PIAAC-based measures. That is, the robustness test assumes that the importance and prevalence of abilities is the same in other countries as in the United States. The robustness test shows the same patterns in terms of AI exposure by occupation, suggesting that it is fine to use the measure linked to PIAAC abilities.

**Figure 5:**
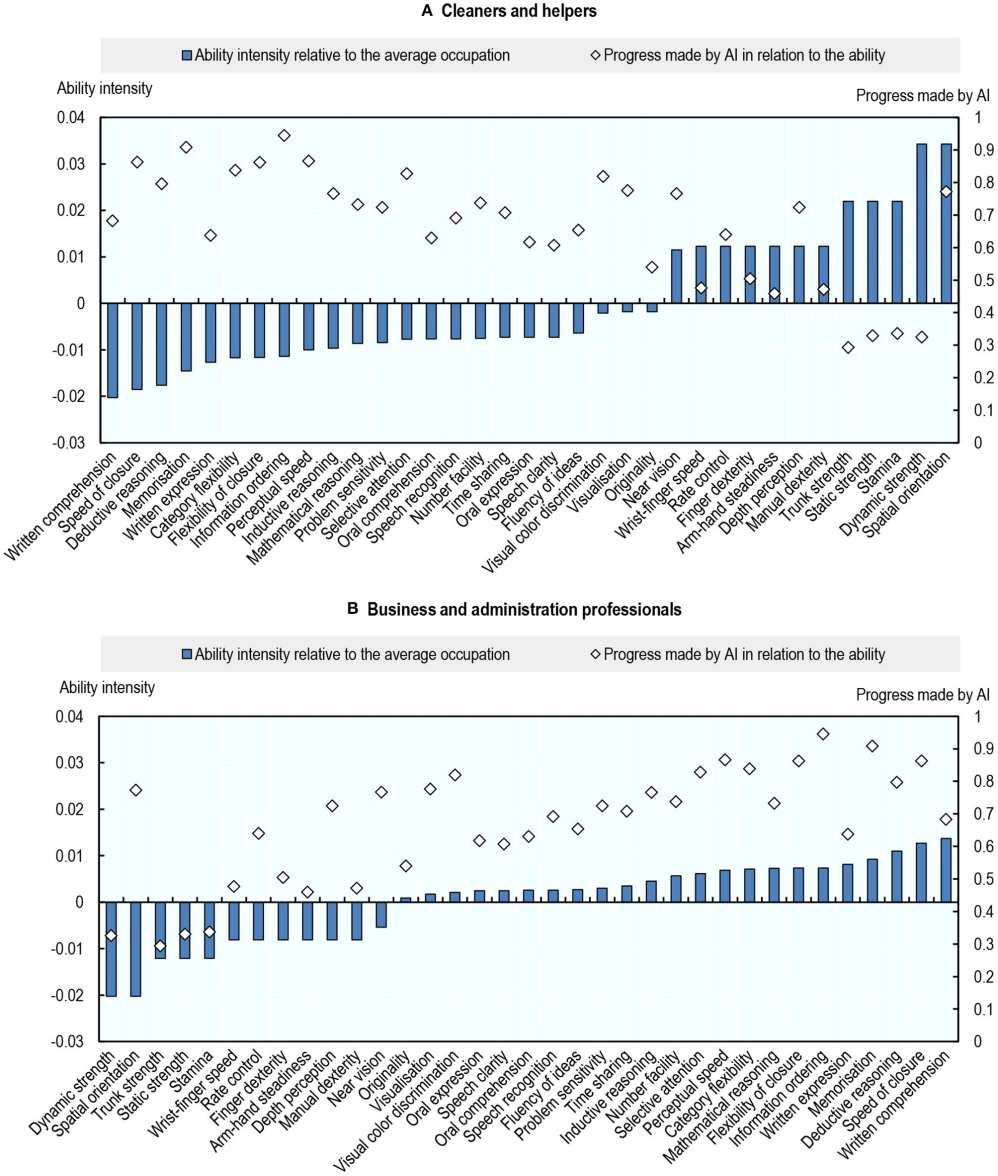
Cross-occupation differences in AI exposure are caused by differences in the intensity of use of abilities. Intensity of use of an ability relative to the average across occupations, and progress made by AI in relation to that ability, 2012. Ability intensity represents the cross-country average frequency of the use of an ability among Cleaner and helpers (top) or Business professionals (bottom) minus the cross-country average frequency of the use of that ability, averaged across the 36 occupations in the sample. Authors' calculations using data from the Programme for the International Assessment of Adult Competencies (PIAAC) and Felten et al. (2019). **(A)** Cleaners and helpers and **(B)** Business and administration professionals.

Cleaners and Helpers, the least exposed occupation according to this measure, have a low score of occupational exposure to AI because they rely less than other workers on cognitive abilities (including those in which AI has made the most progress), whereas they rely more on physical and psychomotor abilities (in which AI has made little progress). Figure 5A illustrates this by plotting the extent to which Cleaners and Helpers use any of the 35 abilities (relative to the average use of that ability across all occupations) against AI progress in that ability. Compared to the average worker, Cleaners and Helpers rely heavily on physical abilities such as dynamic / static/trunk strength and dexterity, areas in which AI has made the least progress in recent years. They rely less than other occupations on abilities with the fastest AI progress, such as information ordering and memorisation. Business Professionals, in contrast, are heavily exposed to AI because they rely more than other workers on cognitive abilities, and less on physical and psychomotor abilities (Figure 5B).

As a robustness check, Figure A A.2 replicates this analysis using O^*^NET scores of “prevalence” and “importance” of abilities within occupations instead of PIAAC-based measures, and it shows the same patterns.

As abilities are the only link between occupations and progress in AI, the occupational exposure measure cannot detect any effects of AI that do not work directly through AI capabilities, for example if AI is employed to make other technologies more efficient. Consider the example of drivers, an occupation often discussed as at-risk of being substituted by AI. Drivers receive a below-average score in the AI occupational exposure measure (see Figure 4). This is because the driving component of autonomous vehicle technologies relies on the physical manipulation of objects, which is in the realm of robotics, not on AI. AI does touch upon some abilities needed to drive a car—such as the ability to plan a route or perceive and distinguish objects at a distance—but the majority of tasks performed when driving a car are physical. AI might well be essential for driverless cars, but mainly by enabling robotic technology, which possesses the physical abilities necessary to drive a vehicle. Thus, this indicator can be seen as isolating the “pure” effects of AI (Felten et al., 2019).

#### Cross-Country Differences in Occupational Exposure to AI

**Figure 6:**
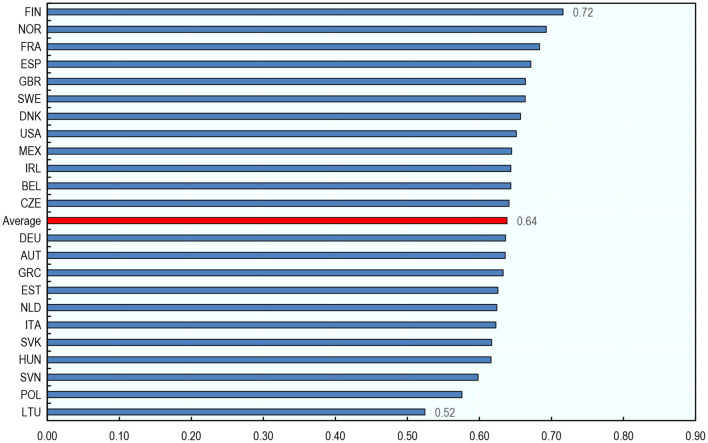
Cross-country differences in exposure to AI for a given occupation are small compared to cross-occupation differences. Average exposure to AI across occupations by country, 2012. The averages presented are unweighted averages across the 36 occupations in the sample. Authors' calculations using data from the Programme for the International Assessment of Adult Competencies (PIAAC) and Felten et al. (2019).

On average, an occupation's exposure to AI varies little across countries—differences across occupations tend to be greater. The average score of AI exposure across occupations ranges from 0.52 (Lithuania) to 0.72 (Finland, Figure 6) among the 23 countries analyzed^33^. By contrast, the average score across countries for the 36 occupations ranges from 0.26 (cleaners and helpers) to 0.87 (business professionals). Even the most exposed cleaners and helpers (in Finland) are only about half as exposed to AI as the least exposed business professionals (in Lithuania) (Figure A A.3). That being said, occupations tend to be slightly more exposed to AI in Northern European countries than in Eastern European ones (Figure 6).

**Figure 7:**
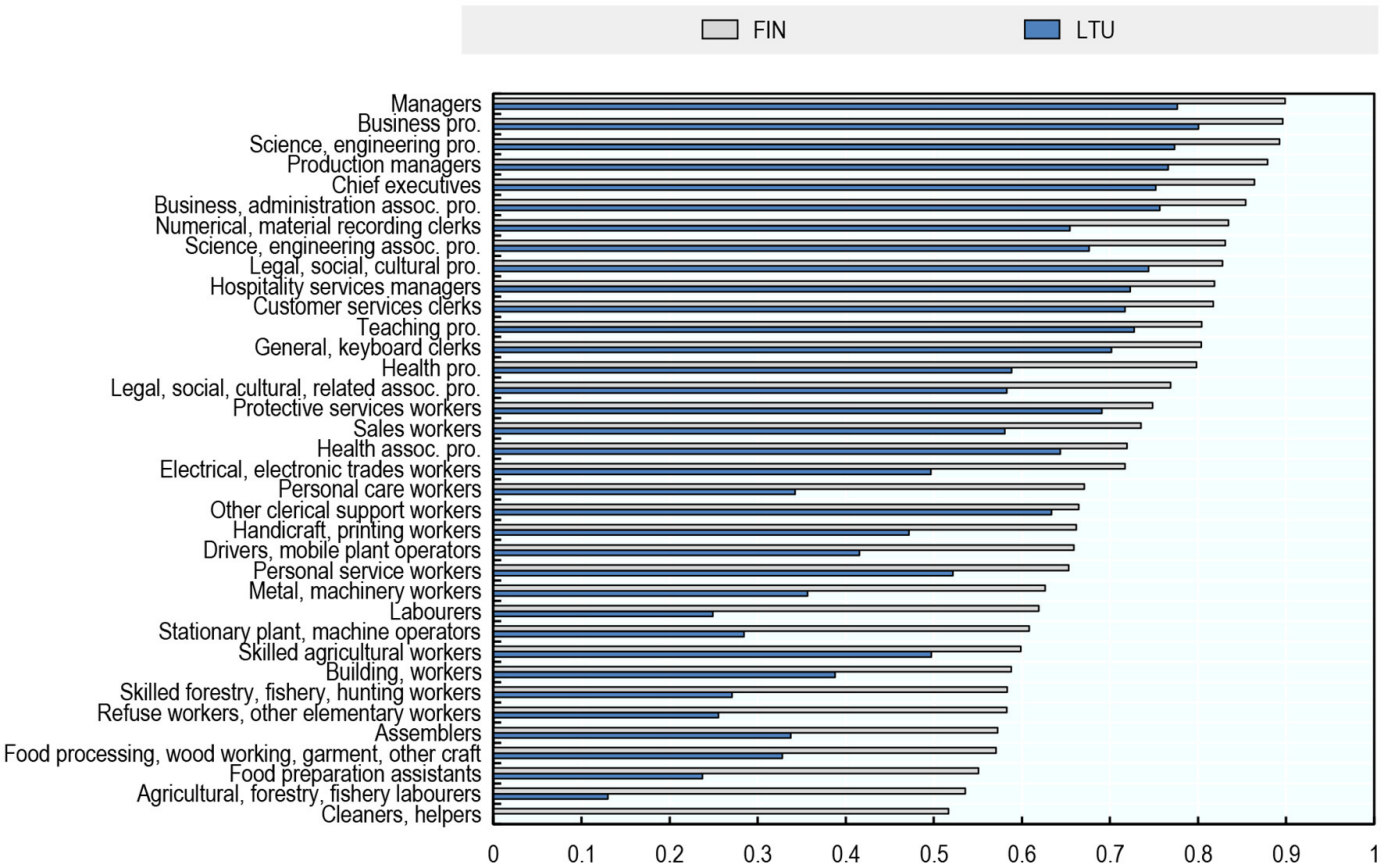
The distribution of AI exposure across occupations is similar in Finland and Lithuania. Exposure to AI, 2012. Authors' calculations using data from the Programme for the International Assessment of Adult Competencies (PIAAC) and Felten et al. (2019).

A different way of showing that AI exposure varies more across occupations than across countries for a given occupation is by contrasting the distribution of exposure to AI across occupations in the most exposed country in the sample (Finland) with that in the least exposed country (Lithuania, Figure 7). The distributions are very similar. In both countries, highly educated white-collar occupations have the highest exposure to AI and non-office-based, physical occupations have the lowest exposure.

Differences in exposure to AI between Finland and Lithuania are greater for occupations in the lower half of the distribution of exposure to AI (Figure 7). For example, Food Preparation Assistants in Finland are more than twice as exposed to AI than food preparation assistants in Lithuania, while the score for Business and Administration Professionals is only 12% higher in Finland than in Lithuania.

**Figure 8:**
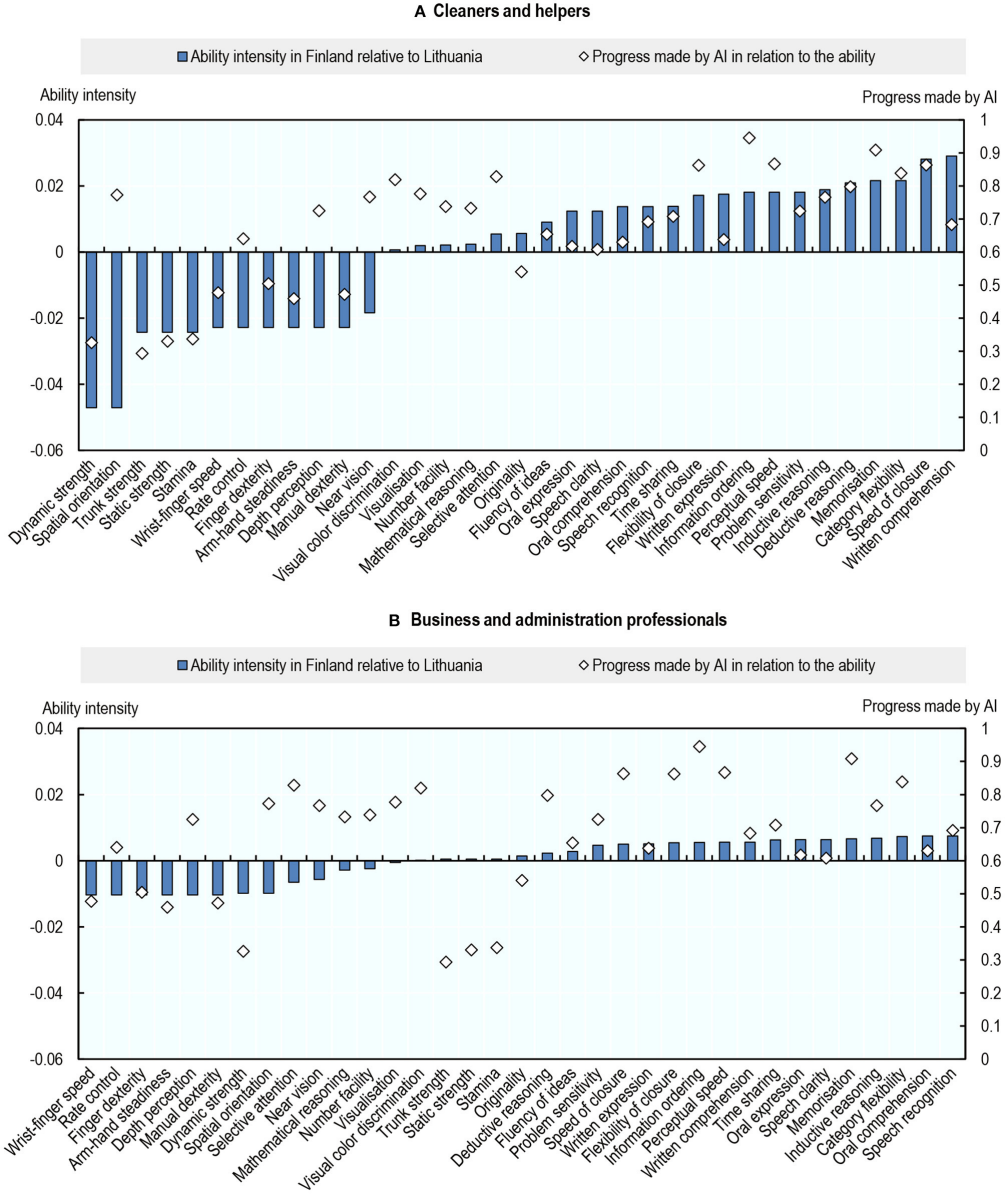
Cross-country differences in occupational AI exposure are caused by differences in the intensity of use of abilities. Intensity of use of an ability in Finland relative to Lithuania and progress made by AI in relation to that ability, 2012. Ability intensity represents the difference in the frequency of the use of an ability among Cleaner and helpers (top) or Business professionals (bottom) between Finland and Lithuania. Authors' calculations using data from the Programme for the International Assessment of Adult Competencies (PIAAC) and Felten et al. (2019). **(A)** Cleaners and helpers and **(B)** Business and administration professionals.

This is because, while occupations across the entire spectrum of exposure to AI rely more on physical than on cognitive abilities in Lithuania than in Finland, this reliance is more pronounced at the low end of the exposure spectrum. Figure 8 illustrates this for the least (Cleaners and Helpers) and the most exposed occupations (Business and Administration Professionals). The top panel displays: (i) the difference in the intensity of use of each ability by Cleaners and Helpers between Finland and Lithuania; and (ii) the progress made by AI in relation to that ability. The bottom panel shows the same for Business and Administration Professionals.

For both occupations, workers in Lithuania tend to rely more on physical and psychomotor abilities (which are little exposed to AI), and less on cognitive abilities, including cognitive abilities in which AI has made the most progress. The differences in the intensity of use of cognitive, physical, and psychomotor abilities between Finland and Lithuania are however greater for Cleaners and Helpers than they are for Business and Administration Professionals (Figure 8). As an example of how cleaners may be more exposed to AI in Finland than in Lithuania, AI navigation tools may help cleaning robots map out their route. They could therefore substitute for cleaners in supervising cleaning robots, especially in countries where cleaning robots are more prevalent (e.g., probably in Finland^34^). More generally, it is likely that cleaners in Finland use more sophisticated equipment and protocols, resulting in a greater reliance on more exposed cognitive abilities. That being said, even in Finland, the least exposed occupation remains Cleaners and Helpers (Figure 7).

Workers in Lithuania may rely more on physical abilities than in Finland because, in 2012, when these ability requirements were measured, technology adoption was more advanced in Finland than in Lithuania. That is, in 2012, technology may have already automated some physical tasks (e.g., cleaning) and created more cognitive tasks (e.g., reading instructions, filling out documentation, supervising cleaning robots) in Finland than in Lithuania, and this might have had a bigger effect on occupations that rely more on physical tasks (like cleaning).

#### Occupational Exposure to AI and Education

**Figure 9:**
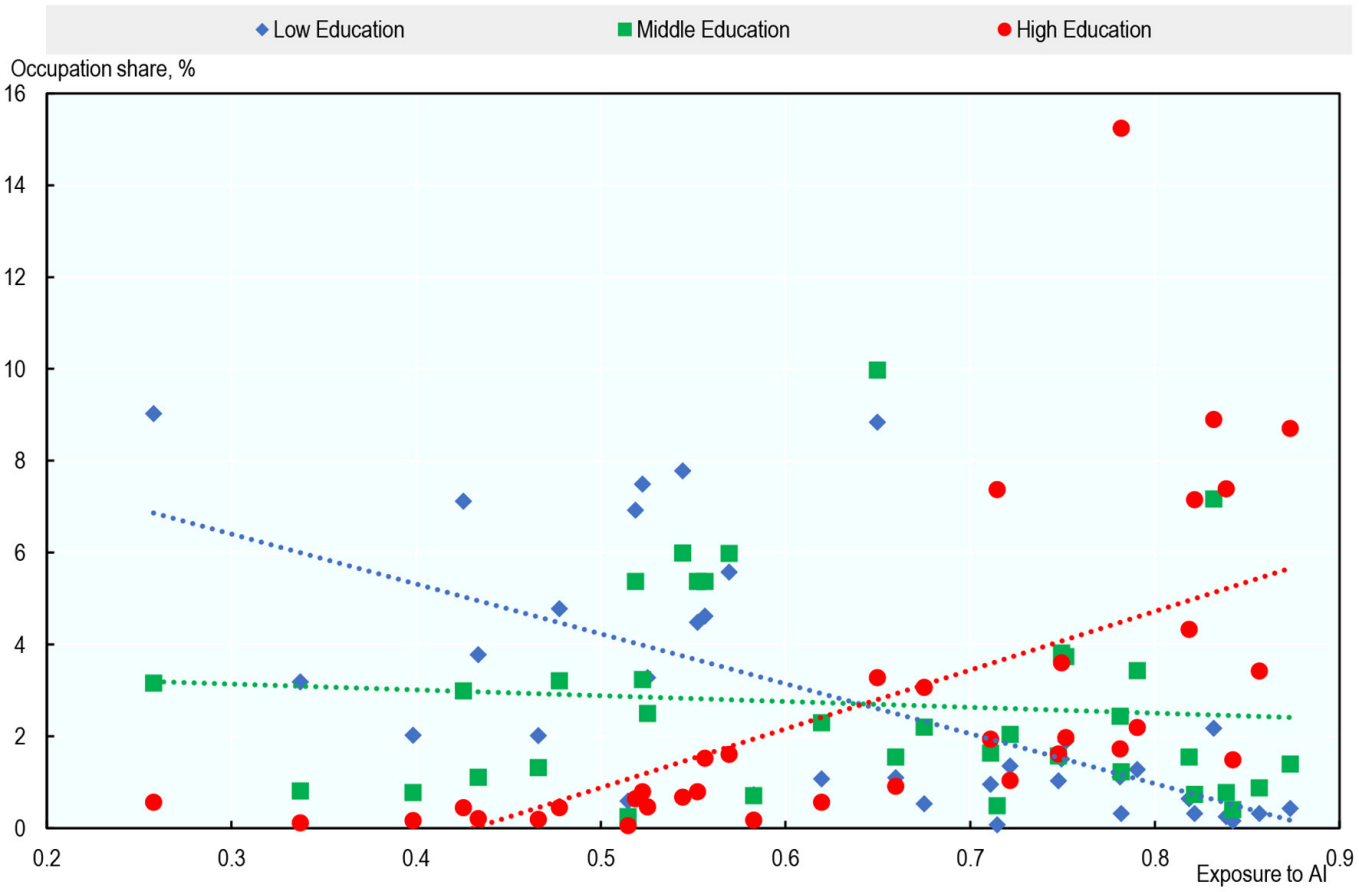
Highly educated workers are disproportionately exposed to AI. Average share of workers with low, medium or high education within occupations vs. average exposure to AI, across countries (2012). For each education group, occupation shares represent the share of workers of that group in a particular occupation. Each dot reports the unweighted average across the 23 countries analyzed of the share of workers with a particular education in an occupation. Authors' calculations using data from the European Union Labor Force Survey (EU-LFS), the Mexican National Survey of Occupation and Employment (ENOE), the US Current Population Survey (US-CPS) PIAAC, and Felten et al. (2019).

Section Occupational Exposure to AI showed that white-collar occupations requiring high levels of formal education are the most exposed to AI, while low-educated physical occupations are the least exposed^35^. Figure 9 confirms this pattern. It shows a clear positive relationship between the share of highly educated workers within an occupation in 2012 and the AI exposure score in that occupation in that year (red line). By contrast, low-educated workers were less likely to work in occupations with high exposure to AI (blue line). The relationship is almost flat for middle-educated workers. In 2012, 82% of highly educated workers were in the most exposed half of occupations, compared to 37% of middle-educated and only 16% of low-educated^36^.

### Labor Market Outcomes

The analysis links occupational exposure to AI to a number of labor market outcomes: employment^37^, average hours worked^38^, the share of part-time workers, and the share of job postings that require AI-related technical skills. This section presents some descriptive statistics on labor market outcomes for the period 2012 and 2019. Two thousand twelve is chosen as the first year for the period of analysis because it ensures consistency with the measure of occupational exposure to AI, for two reasons. First, the measure of exposure to AI is based on the task composition of occupations in 2012 for most countries^39^. Second, progress in AI applications is measured over the period 2010–2015. As a result, AI, as proxied by the occupational AI exposure indicator, could affect the labor market starting from 2010 and fully from 2015 onwards. Starting in 2012 provides a long enough observation period, while closely tracking the measure of recent developments in AI.

#### Employment and Working Hours

**Figure 10:**
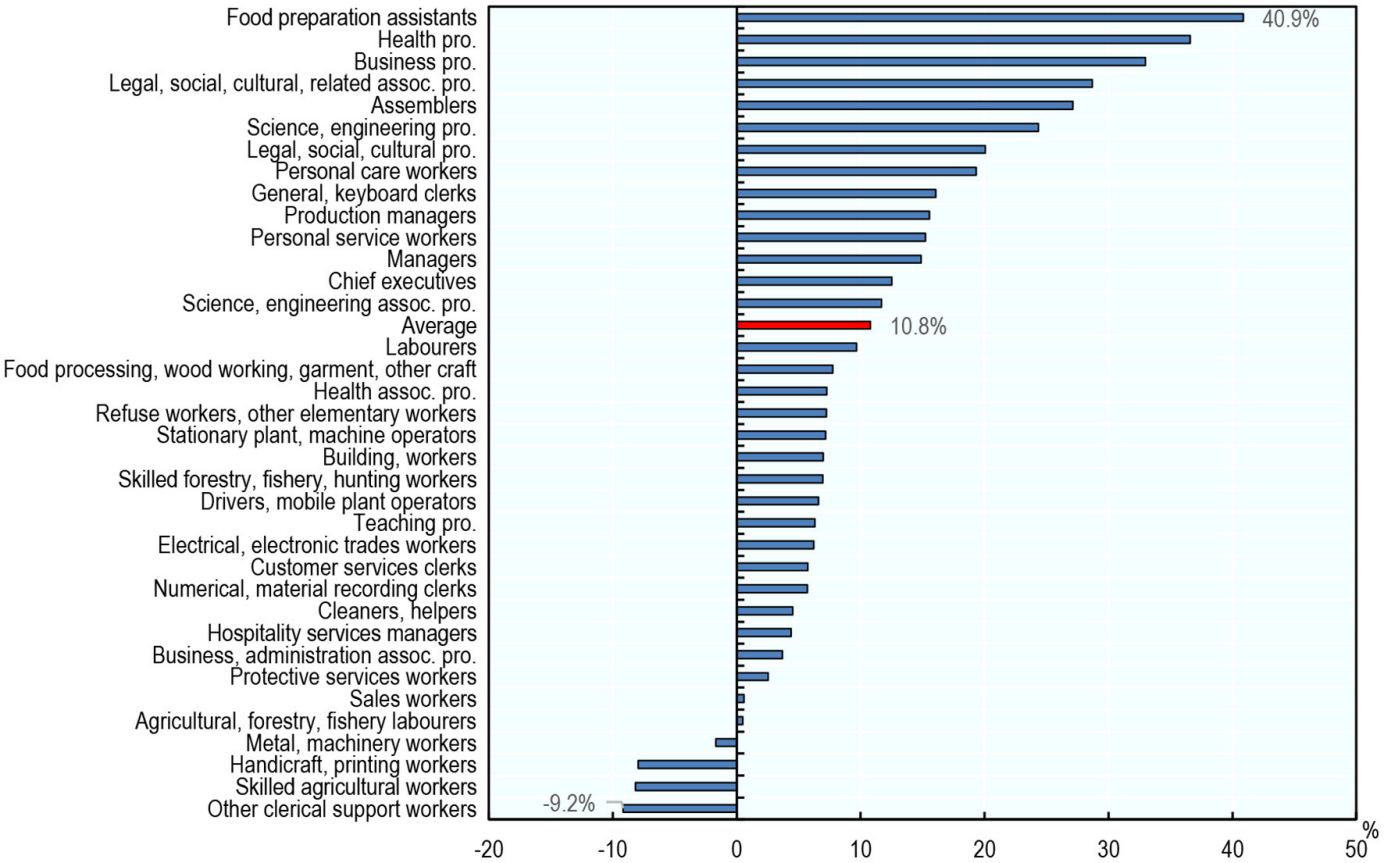
Employment has grown in most occupations between 2012 and 2019. Average percentage change in employment level across countries by occupation, 2012–2019. Occupations are classified using two-digit ISCO-08. The averages presented are unweighted averages across the 23 countries analyzed. Source: ENOE, EU-LFS, and US-CPS.

Overall, in most occupations and on average across the 23 countries, employment grew between 2012 and 2019, a period that coincides with the economic recovery from the global financial crisis. Employment grew by 10.8% on average across all occupations and countries in the sample (Figure 10). Average employment growth was negative for only four occupations: Other Clerical Support Workers (−9.2%), Skilled Agricultural Workers (−8.2%), Handicraft and Printing Workers (−7.9%), and Metal and Machinery Workers (-1.7%).

**Figure 11:**
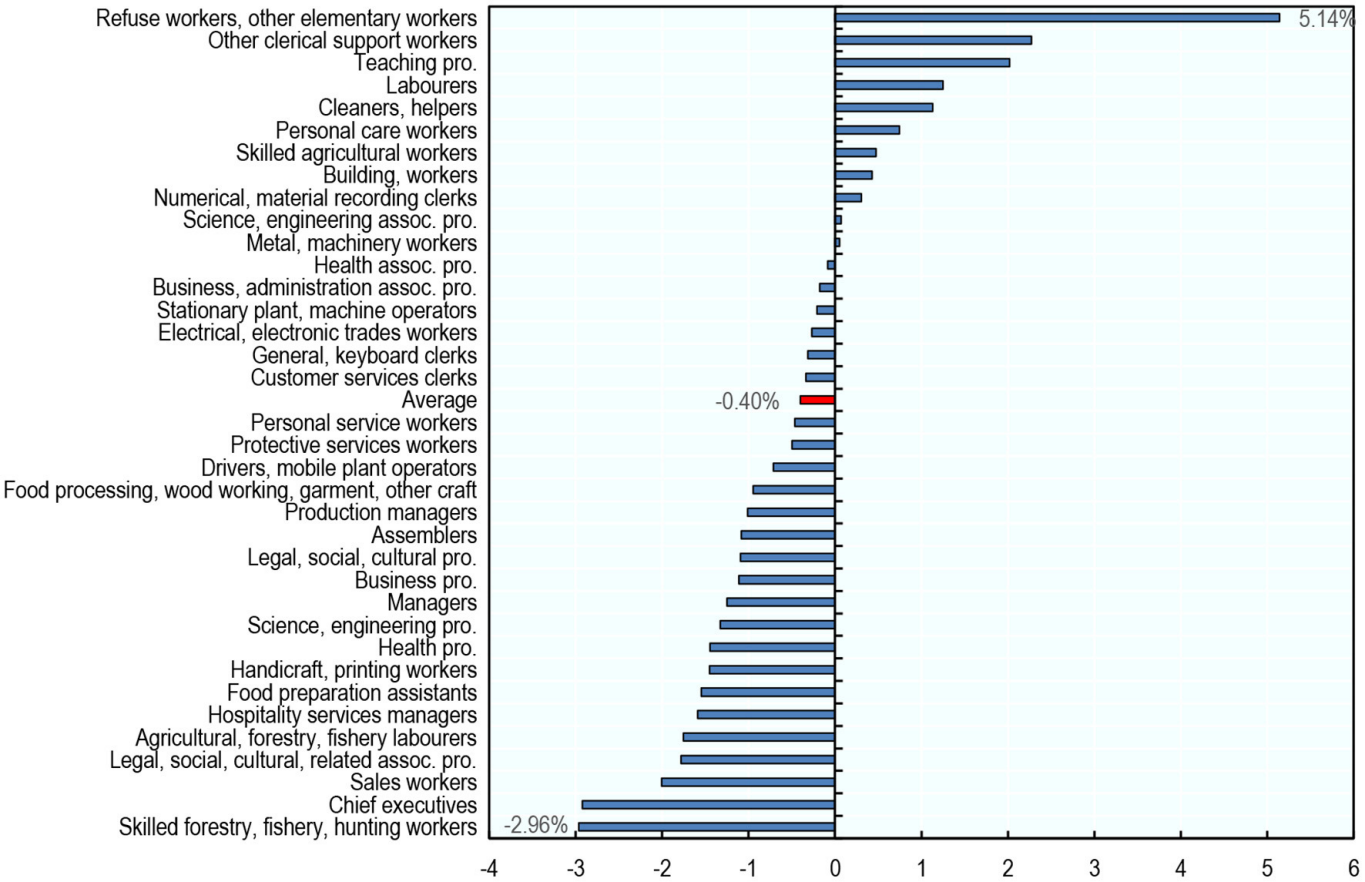
Average usual working hours have decreased in most occupations between 2012 and 2019. Average percentage change in average usual weekly hours across countries by occupation, 2012–2019. Occupations are classified using two-digit ISCO-08. The averages presented are unweighted averages across the 22 countries analyzed (Mexico is excluded from the analysis of working time due to data availability). Usual weekly working hours by country-occupation cell are calculated by taking the average across individuals within that cell. Source: ENOE, EU-LFS, and US-CPS.

By contrast, average usual weekly hours declined by 0.40% (equivalent to 9 min per week^40^ average over the same period (Figure 11)^41^. On average across countries, working hours declined in most occupations. Occupations with the largest drops in working hours include (but are not limited to) occupations that most often use part-time employment, such as Sales Workers (−2.0%); Legal, Social, Cultural Related Associate Professionals (−1.8%); and Agricultural, Forestry, Fishery Laborers (−1.8%).

#### Job Postings That Require AI Skills

Beyond its effects on job quantity, AI may transform occupations by changing their task composition, as certain tasks are automated and workers are increasingly expected to focus on other tasks. This may result in a higher demand for AI-related technical skills as workers interact with these new technologies. However, it is not necessarily the case that working with AI requires technical AI skills. For example, a translator using an AI translation tool does not necessarily need any AI technical skills.

This section looks at the share of job postings that require AI-related technical skills (*AI skills*) by occupation using job postings data from Burning Glass Technologies^42^ for the United Kingdom and the United States^43^^.^ AI-related technical skills are identified based on the list provided in Acemoglu et al. (2020)^44^.

**Figure 12:**
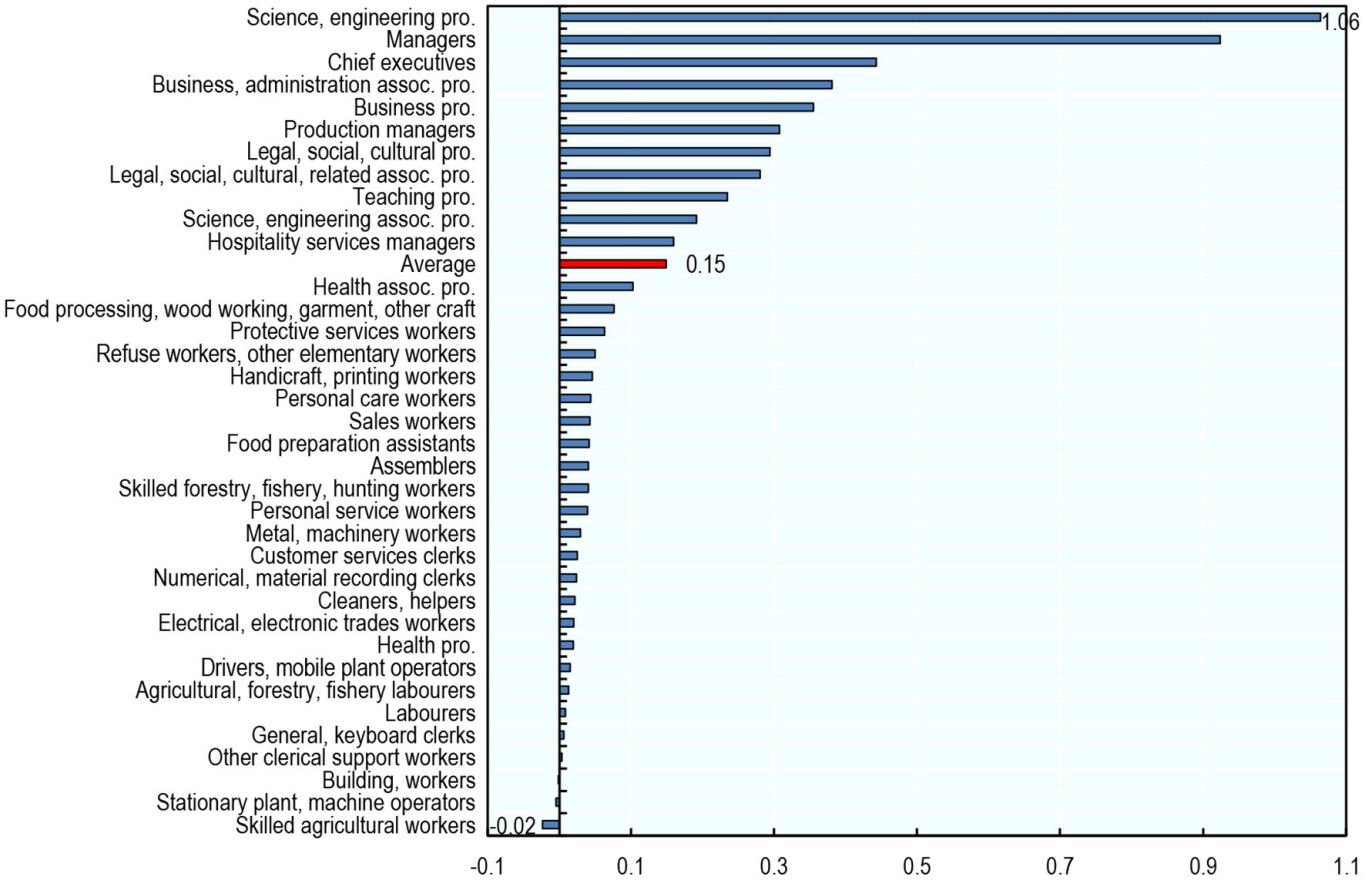
Nearly all occupations have increasingly demanded AI skills between 2012 and 2019 in the United States. Percentage point* change in the share of job postings that require AI skills, 2012–2019, USA. The share of job postings that require AI skills in an occupation is the number of job postings requiring such skills in that occupation divided by the total number of job postings in that same occupation. *Percentage point changes are preferred over percentage changes because the share of job postings that require AI skills is equal to zero in some occupations in 2012. Source: Burning Glass Technologies.

In the United States, the share of job postings requiring AI skills has increased in almost all occupations between 2012 and 2019 (Figure 12). Science and Engineering Professionals experienced the largest increase, but growth was also substantial for Managers, Chief Executives, Business and Administration Professionals, and Legal, Social, Cultural Professionals. That being said, the share of job postings that require AI skills remains very low overall, with an average across occupations of 0.24% in 2019 (against 0.10% in 2012). These orders of magnitude are in line with Acemoglu et al. (2020) and Squicciarini and Nachtigall (2021).

## Results

This section looks at the link between an occupation's exposure to AI in 2012 and changes in employment, working hours, and the demand for AI-related technical skills between 2012 and 2019. Exposure to AI appears to be associated with greater employment growth in occupations where computer use is high, and larger reductions in hours worked in occupations where computer use is low. So, even though AI may substitute for workers in certain tasks, it also appears to create job opportunities in occupations that require digital skills. In addition, there is some evidence that greater exposure to AI is associated with greater increase in demand for AI-related technical skills (such as natural language processing, machine translation, or image recognition) in occupations where computer use is high. However, as the share of jobs requiring AI skills remains very small, this increase in jobs requiring AI skills cannot account for the additional employment growth observed in computer-intensive occupations that are exposed to AI.

### Empirical Strategy

The analysis links changes in employment levels within occupations and across countries to AI exposure^45^. The regression equation is the following:


(1) $$\begin{array}{l} {\text{Y}}_{\text{ij}}=\alpha_{\text{j}}+\beta \text{ A}{\text{I}}_{\text{ij}}+{\gamma \text{ X}}_{\text{ij}}\;+{\text{u}}_{\text{ij}} \end{array}$$

where *Y*_ij_ is the percentage change in the number of workers (both dependent employees and self-employed) in occupation i in country j over the period 2012–2019^46^; *AI*_ij_ is the index of exposure to AI for occupation i in country j as measured in 2012; *X*_ij_ is a vector of controls including exposure to other technological advances (software and industrial robots), offshorability, exposure to international trade, and 1-digit occupational ISCO dummies; α_j_ are country fixed effects; and *u*_ij_ is the error term. The coefficient of interest β captures the link between exposure to AI and changes in employment. The inclusion of country fixed effects means that the analysis only exploits within-country variation in AI exposure to estimate the parameter of interest. The specifications that include 1-digit occupational dummies only exploit variation within broad occupational groups, thereby controlling for any factors that are constant across these groups.

To control for the effect of non-AI technologies, the analysis includes measures of exposure to software and industrial robots developed by Webb (2020) based on the overlap between the text of job descriptions provided in the O^*^NET database and the text of patents in the fields corresponding to each of these technologies^47^. Offshoring is proxied by an index of offshorability developed by Firpo et al. (2011) and made available by Autor and Dorn (2013), which measures the potential offshoring of job tasks using the average between the two variables “Face-to-Face Contact” and “On-Site Job” that Firpo et al. (2011) derive from the O^*^NET database^48^. This measure captures the extent to which an occupation requires direct interpersonal interaction or proximity to a specific work location^49^.

The three above indices are occupation-level task-based measures derived from the O^*^NET database for the United States; this analysis uses those measures for all 23 countries, assuming that the cross-occupation distribution of these indicators is similar across countries^50^. Exposure to international trade is proxied by the share of employment within occupations that is in tradable sectors^51^. These shares are derived from the European Union Labor Force Survey (EU-LFS), the Mexican National Survey of Occupation and Employment (ENOE), the US Current Population Survey (US-CPS).

### Exposure to AI and Employment: A Positive Relationship in Occupations Where Computer Use Is High

As discussed in Section Introduction, the effect of exposure to AI on employment is theoretically ambiguous. On the one hand, employment may fall as tasks are automated (substitution effect). On the other hand, productivity gains may increase labor demand (productivity effect) (Acemoglu and Restrepo, 2019a,b; Bessen, 2019; Lane and Saint-Martin, 2021)^52^. The labor market impact of AI on a given occupation is likely to depend on the task composition of that occupation—the prevalence of high-value added tasks that AI cannot automate (e.g., tasks that require creativity or social intelligence) or the extent to which the occupation already uses other digital technologies [since AI applications are often similar to software in their use, workers with digital skills may find it easier to use AI effectively (Felten et al., 2019)]. Therefore, the following analysis will not only look at the entire sample of occupation-country cells, but will also split the sample according to what people do in these occupations and countries.

In particular, the level of computer use within an occupation is proxied by the share of workers reporting the use of a computer at work in that occupation, calculated for each of the 23 countries in the sample. It is based on individuals' answers to the question “Do you use a computer in your job?,” taken from the Survey of Adult Skills (PIAAC). Occupation-country cells are then classified into three categories of computer use (low, medium, and high), where the terciles are calculated based on the full sample of occupation-country cells^53^. Another classification used is the country-invariant classification developed by Goos et al. (2014), which classifies occupations based on their average wage relying on European Community Household Panel (ECHP) data. For example, occupations with an average wage in the middle of the occupation-wage distribution would be classified in the middle with respect to this classification^54^. Finally, the prevalence of creative and social tasks is derived from PIAAC data. PIAAC data include the frequency with which a number of tasks are performed at the individual level. Respondents' self-assessment are based on a 5-point scale ranging from “Never” to “Every day.” This information is used to measure the average frequency with which workers in each occupation perform creative or social tasks, and this is done separately for each country^55^.

While employment grew faster in occupations more exposed to AI, this relationship is not robust. There is stronger evidence that AI exposure is positively related to employment growth in occupations where computer use is high. Table 2 displays the results of regression equation (1) without controls. When looking at the entire sample, the coefficient on AI exposure is both positive and statistically significant (Column 1), but the coefficient is no longer statistically significant as soon as any of the controls described in Section Empirical Strategy are included (with the exception of offshorability)^56^. When the sample is split by level of computer use (low, medium, high), the coefficient on AI exposure remains positive and statistically significant only for the subsample where computer use is high (Columns 2–4). It remains so after successive inclusion of controls for international trade (i.e., shares of workers in tradable sectors), offshorability, exposure to other technological advances (software and industrial robots) and 1-digit occupational dummies (Table 3)^57^. In occupations where computer use is high, a one standard deviation increase in AI exposure is associated with 5.7 percentage points higher employment growth (Table 2, Column 4)^58^.

**Table 2:** Exposure to AI is positively associated with employment growth in occupations where computer use is high.

	**(1)** **All occupations**	**(2)** **Low computer use**	**(3)** **Medium computer use**	**(4)** **High computer use**
Exposure to AI	13.3^**^	−3.7	8.3	85.7^**^
	(6.4)	(13.2)	(18.4)	(36.5)
Country FEs	Yes	Yes	Yes	Yes
Observations	822	274	274	274
R-squared	0.058	0.127	0.172	0.098

*Dependent variable: 2012–2019% change in employment level*.

*Robust standard errors in parentheses*.

^***^p < 0.01,

** *p < 0.05*,

^*^p < 0.1.

*Each observation is a country-occupation cell*.

*Each column shows the results of regression equation (1) applied to one of the subsamples obtained by splitting the overall sample by level of computer use. Occupation-country cells are classified into low, medium or high computer use by tercile of computer use applied across the full sample of occupation-country cells*.

*Source: Authors' calculations using data from ENOE, EU-LFS, US-CPS, PIAAC, and Felten et al. (2019)*.

**Table 3:** The relationship between exposure to AI and employment growth is robust to the inclusion of a number of controls.

	**(1)**	**(2)**	**(3)**	**(4)**	**(5)**
**High computer use occupations**					
Exposure to AI	85.7^**^	94.4^***^	137.7^***^	135.4^***^	144.6^**^
	(36.5)	(34.7)	(36.5)	(40.6)	(62.6)
Share of tradable sectors		−0.143	−0.0120	−0.00931	0.157
		(0.151)	(0.145)	(0.166)	(0.256)
Offshorability			−7.4^**^	−7.4^***^	−9.7^**^
			(2.9)	(2.8)	(4.6)
Exposure to softwares				0.0103	0.00429
				(0.190)	(0.253)
Exposure to robots				−0.0241	0.258
				(0.280)	(0.341)
1-digit occupation FEs	No	No	No	No	Yes
Country FEs	Yes	Yes	Yes	Yes	Yes
Observations	274	274	274	274	274
R-squared	0.098	0.101	0.127	0.127	0.173

*Dependent variable: 2012–2019 % change in employment level. Robust standard errors in parentheses*.

*** *p < 0.01*,

** *p < 0.05*,

^*^p < 0.1.

*Each observation is a country-occupation cell. The sample is restricted to occupations with high computer use. Occupation-country cells are classified into low, medium or high computer use by tercile of computer use applied across the full sample of occupation-country cells. Offshorability is an occupation-level measure from Autor and Dorn (2013) based on data from the United States. Exposure to software and exposure to robots are occupation-level measures developed by Webb (2020) based on data from the United States. The share of tradable sector represents the 2012 share of workers in the country-occupation cell working in agriculture, industry, and financial and insurance activities. Source: Authors' calculations using data from ENOE, EU-LFS, US-CPS, PIAAC, Autor and Dorn (2013), Felten et al. (2019), and Webb (2020)*.

By contrast, the average wage level of the occupation or the prevalence of creative or social tasks matter little in the link between exposure to AI and employment growth. Table A A.1 in Appendix shows the results obtained when replicating the analysis on the subsamples obtained by splitting the overall sample by average wage level, prevalence of creative tasks, or prevalence of social tasks. All coefficients on exposure to AI remain positive, but are weakly statistically significant and of lower magnitude than those obtained on the subsample of occupations where computer use is high (Table 3).

As a robustness check, Table A A.2 in the Appendix replicates the analysis in Table 2 using the score of exposure to AI obtained when using O^*^NET scores of “prevalence” and “importance” of abilities within occupations instead of PIAAC-based measures. The results remain unchanged. Table A A.3 replicates the analysis using the alternative indicators of exposure to AI constructed by Webb (2020) and Tolan et al. (2021), described in Section What Do These Indicators Measure?^59^ While the Webb (2020) indicator confirms the positive relationship between employment growth and exposure to AI in occupations where computer use is high, the coefficient obtained with the Tolan et al. (2021) indicator is positive but not statistically significant. This could be due to the fact that the Tolan et al. (2021) indicator reflects different aspects of AI advances, as it focuses more on cognitive abilities and is based on research intensity rather than on measures of progress in AI applications.

**Figure 13:**
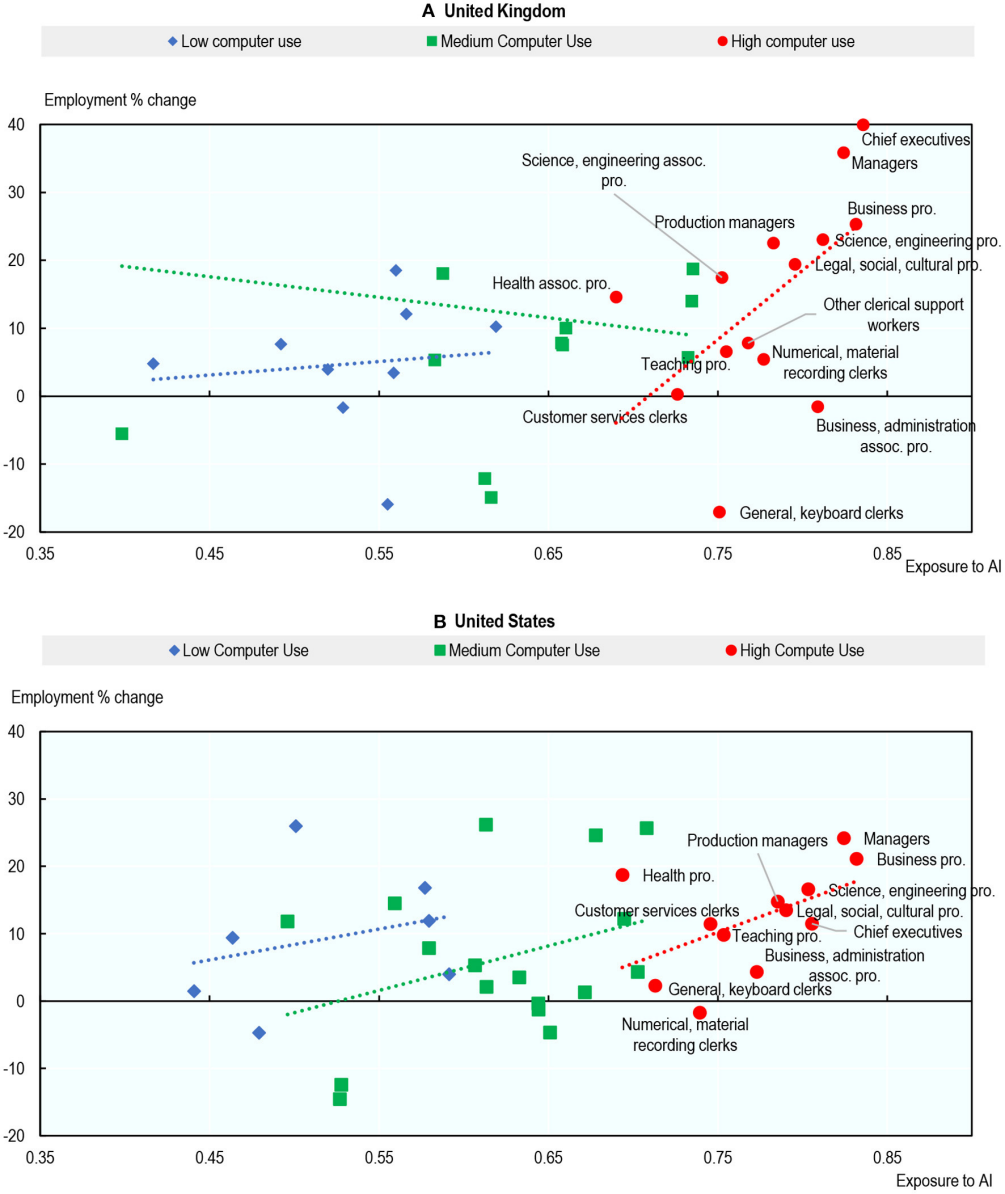
Exposure to AI is associated with higher employment growth in occupations where computer use is high. Percentage change in employment level (2012–2019) and exposure to AI (2012). Occupations are classified using two-digit ISCO-08. Not all occupations have marker labels due to space constraints. Skilled forestry, fishery, hunting workers excluded from **(A)** for readability reasons. Occupation-country cells are classified into low, medium or high computer use by tercile of computer use applied across the full sample of occupation-country cells. Source: Authors' calculations using data from EU-LFS, US-CPS, PIAAC, and Felten et al. (2019). **(A)** United Kingdom and **(B)** United States.

The examples of the United Kingdom and the United States illustrate these findings clearly^60^. Figure 13 shows the percentage change in employment from 2012 to 2019 for each occupation against that occupation's exposure to AI in 2012, both in the United Kingdom (Figure 13A) and the United States (Figure 13B). Occupations are classified according to their level of computer use. The relationship between exposure to AI and employment growth within computer use groups is generally positive, but the correlation is stronger in occupations where computer use is high. For occupations with high computer use, the most exposed occupations tend to have experienced higher employment growth between 2012 and 2019: Business Professionals; Legal, Social and Cultural Professionals; Managers; and Science & Engineering Professionals. AI applications relevant to these occupations include: identifying investment opportunities, optimizing production in manufacturing plants, identifying problems on assembly lines, analyzing and filtering recorded job interviews, and translation. In contrast, high computer-use occupations with low or negative employment growth were occupations with relatively low exposure to AI, such as clerical workers and teaching professionals.

While further research is needed to test the causal nature of these patterns and to identify the exact mechanism behind them, it is possible that a high level of digital skills (as proxied by computer use) indicates a greater ability of workers to adapt to and use new technologies at work and, hence, to reap the benefits that these technologies bring. If AI allows these workers to interact with AI and to substantially increase their productivity and/or the quality of their output, this may, under certain conditions, lead to an increase in demand for their labor^61^.

### Exposure to AI and Working Time: A Negative Relationship Among Occupations Where Computer Use Is Low

This subsection extends the analysis by shifting the focus from the number of working individuals (extensive margin of employment) to how much these individuals work (intensive margin).

In general, the higher the level of exposure to AI in an occupation, the greater the drop in average hours worked over the period 2012–2019; and this relationship is particularly marked in occupations where computer use is low. Column (1) of Table 4 presents the results of regression equation (1) using the percentage change in average usual weekly working hours as the variable of interest. The statistically significant and negative coefficient on exposure to AI highlights a negative relationship across the entire sample. Splitting the sample by computer use category shows that this relationship is stronger among occupations with lower computer use (Column 2–4). The size of the coefficients in Column 2 indicates that, within countries and across occupations with low computer use, a one standard deviation increase in exposure to AI is associated with a 0.60 percentage point greater drop in usual weekly working hours^62^ (equivalent to 13 min per week)^63^. Columns 1–4 of Table 5 show that the result is robust to the successive inclusion of controls for international trade, offshorability, and exposure to other technologies. However, the coefficient on exposure to AI loses statistical significance when controlling for 1 digit occupational dummies (Table 5, Column 5), which could stem from attenuation bias, as measurement errors may be significant relative to the variation in actual exposure within the 1 digit occupation groups^64^.

**Table 4:** Exposure to AI is negatively associated with the growth in average working hours in occupations where computer use is low.

	**(1)**	**(2)**	**(3)**	**(4)**	**(5)**	**(6)**	**(7)**	**(8)**
	**Dependent variable: 2012–2019% change in working hours**				**Dependent variable: 2012–2019% change in part-time employment**			
	**All occupations**	**Low computer use**	**Medium computer use**	**High computer use**	**All occupations**	**Low computer use**	**Medium computer use**	**High computer use**
Exposure to AI	−2.7^***^	−4.8^**^	−4.1	−3.2	14.9	56.6^**^	−37.6	2.4
	(0.9)	(2.3)	(3.1)	(3.1)	(10.0)	(24.7)	(94.1)	(53.7)
Country FEs	Yes	Yes	Yes	Yes	Yes	Yes	Yes	Yes
Observations	781	252	261	268	781	252	261	268
R-squared	0.143	0.133	0.209	0.304	0.143	0.206	0.193	0.211

*Robust standard errors in parentheses*.

*** *p < 0.01*,

** *p < 0.05*,

^*^p < 0.1.

*Each observation is a country-occupation cell. Each column shows the results of regression equation (1) applied to one of the subsamples obtained by splitting the overall sample by level of computer use. Occupation-country cells are classified into low, medium or high computer use by tercile of computer use applied across the full sample of occupation-country cells. In columns 1–4, the dependent variable is the percentage change in average usual weekly working hours. In columns 5–8, the dependent variable is the percentage change in the share of part-time workers. Mexico is excluded from the analysis of working time due to data availability*.

*Source: Authors' calculations using data from EU-LFS, US-CPS, PIAAC, and Felten et al. (2019)*.

**Table 5:** The relationship between exposure to AI and growth in average working hours is robust to the inclusion of a number of controls.

	**(1)**	**(2)**	**(3)**	**(4)**	**(5)**	**(6)**	**(7)**	**(8)**	**(9)**	**(10)**
	**Dependent variable: 2012–2019% change in working hours**					**Dependent variable: 2012–2019% change in part-time employment**				
	**Low computer use occupations**					**Low computer use occupations**				
Exposure to AI	−4.8^**^	−4.9^**^	−9.2^***^	−9.2^**^	−7.2	56.6^**^	56.6^**^	49.4^**^	53.0	23.5
	(2.3)	(2.3)	(3.2)	(4.0)	(4.6)	(24.7)	(24.7)	(24.5)	(35.3)	(41.7)
Share of tradable sectors		−0.0148	−0.0194^*^	−0.0267^**^	−0.0222		0.0135	0.00582	−0.00142	−0.0721
		(0.0111)	(0.0116)	(0.0133)	(0.0176)		(0.113)	(0.124)	(0.139)	(0.167)
Offshorability			−1.4^**^	−0.970	−0.887			−2.4	−1.6	−2.9
			(0.7)	(0.8)	(0.9)			(8.4)	(11.9)	(12.8)
Exposure to softwares				0.0289	0.0350				0.0358	−0.0567
				(0.0263)	(0.0300)				(0.314)	(0.376)
Exposure to robots				−0.0270	−0.0364				0.0151	0.00943
				(0.0385)	(0.0619)				(0.447)	(0.744)
1-digit occupation FEs	No	No	No	No	Yes	No	No	No	No	Yes
Country FEs	Yes	Yes	Yes	Yes	Yes	Yes	Yes	Yes	Yes	Yes
Observations	252	252	252	252	252	252	252	252	252	252
R-squared	0.133	0.141	0.157	0.161	0.166	0.206	0.206	0.207	0.207	0.214

*Robust standard errors in parentheses*.

*** *p < 0.01*,

** *p < 0.05*,

* *p < 0.1*.

*Each observation is a country-occupation cell. The sample is restricted to occupations with low computer use. Occupation-country cells are classified into low, medium or high computer use by tercile of computer use applied across the full sample of occupation-country cells. In columns 1–4, the dependent variable is the percentage change in average usual weekly working hours. In columns 5–8, the dependent variable is the percentage change in the share of part-time workers. Offshorability is an occupation-level measure from Autor and Dorn (2013) based on data from the United States. Exposure to software and exposure to robots are occupation-level measures developed by Webb (2020) based on data from the United States. The share of tradable sector represents the 2012 share of workers in the country-occupation cell working in: agriculture, industry, and financial and insurance activities. Mexico is excluded from the analysis of working time due to data availability. Source: Authors' calculations using data from EU-LFS, US-CPS, PIAAC, Autor and Dorn (2013), Felten et al. (2019), and Webb (2020)*.

The relationship between exposure to AI and the drop in average hours worked was driven by part-time employment^65^. Columns 5–8 of Table 4 replicate the analysis in Columns 1–4 using the change in the occupation-level share of part-time workers as the variable of interest. The results are consistent with those in columns 2–4: the coefficient on exposure to AI is positive and statistically significant only for the subsample of occupations where computer use is low (Columns 6–8). The coefficient remains statistically significant and positive when controlling for international trade and offshorability, but loses statistical significance when controlling for exposure to other technological advances and 1-digit occupational dummies (Table 5, columns 6–10)^66^. The results hold when replacing the share of part-time workers with the share of involuntary part-time workers^67^ (Table A A.7), suggesting that the additional decline in working hours among low computer use occupations that are exposed to AI is not a voluntary choice by workers.

**Figure 14:**
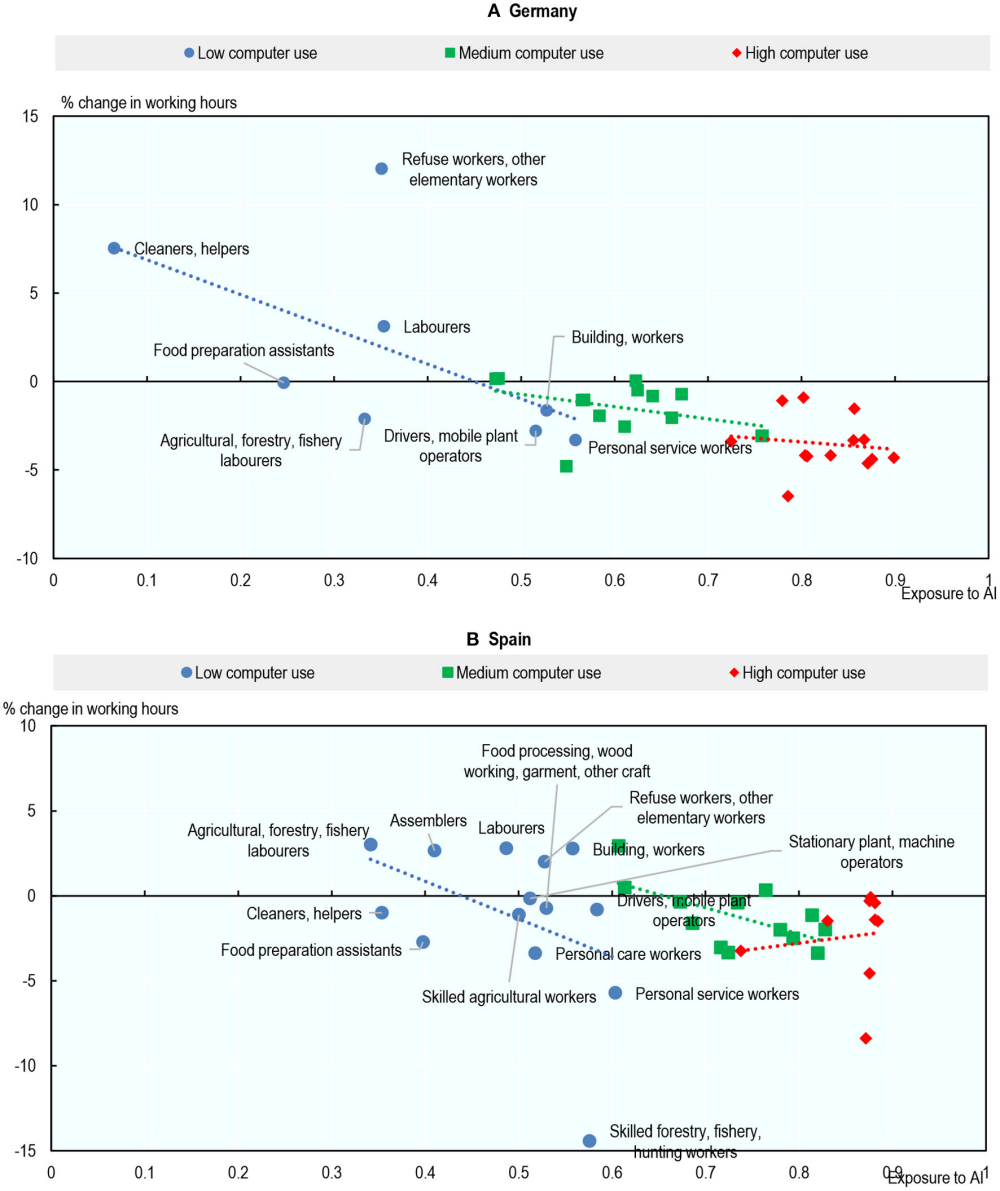
In occupations where computer use is low, exposure to AI is negatively associated with the growth in average working hours. Percentage change in average usual working hour (2012–2019) and exposure to AI (2012). Occupations are classified using two-digit ISCO-08. Not all occupations have marker labels due to space constraints. Occupation-country cells are classified into low, medium or high computer use by tercile of computer use applied across the full sample of occupation-country cells. Source: Author' calculations using data from EU-LFS, PIAAC, and Felten et al. (2019). **(A)** Germany and **(B)** Spain.

The examples of Germany and Spain provide a good illustration of these results^68^. Figure 14 shows the percentage change in average usual weekly working hours from 2012 to 2019 for each occupation against that occupation's exposure to AI, both in Germany (Figure 14A) and in Spain (Figure 14B). As before, occupations are classified according to their degree of computer use (low, medium, high). In both countries, there is a clear negative relationship between exposure to AI and the change in working hours among occupations where computer use is low. In particular, within the low computer use category, most occupations with negative growth in working hours are relatively exposed to AI. These occupations include: Drivers and Mobile Plant Operators, Personal Service Workers, and Skilled Agricultural Workers. AI applications relevant to these occupations include route optimisation for drivers, personalized chatbots and demand forecasting in the tourism industry^69^, or the use of computer vision in the agricultural sector to identify plants that need special attention. By contrast, low computer use occupations with the strongest growth in working hours are generally less exposed to AI. This is for example the case for Laborers (which includes laborers in transport and storage, manufacturing, or mining and construction).

Again, while further research is required, a lack of digital skills may mean that workers are not able to interact efficiently with AI and thus cannot reap all potential benefits of the technology. The substitution effect of AI in those occupations therefore appears to outweigh the productivity effect, resulting in reduced working hours, possibly as a result of more involuntary part-time employment. However, these results remain suggestive, as they are not robust to the inclusion of the full set of controls and the use of alternative indicators of exposure to AI.

### Exposure to AI and Demand for AI-Related Technical Skills: A Weak but Positive Relationship Among Occupations Where Computer Use Is High

Beyond its effects on employment, AI may also transform occupations as workers are increasingly expected to interact with the technology. This may result in a higher demand for AI-related technical skills in affected occupations, although it is not necessarily the case that working with AI requires technical AI skills.

**Figure 15:**
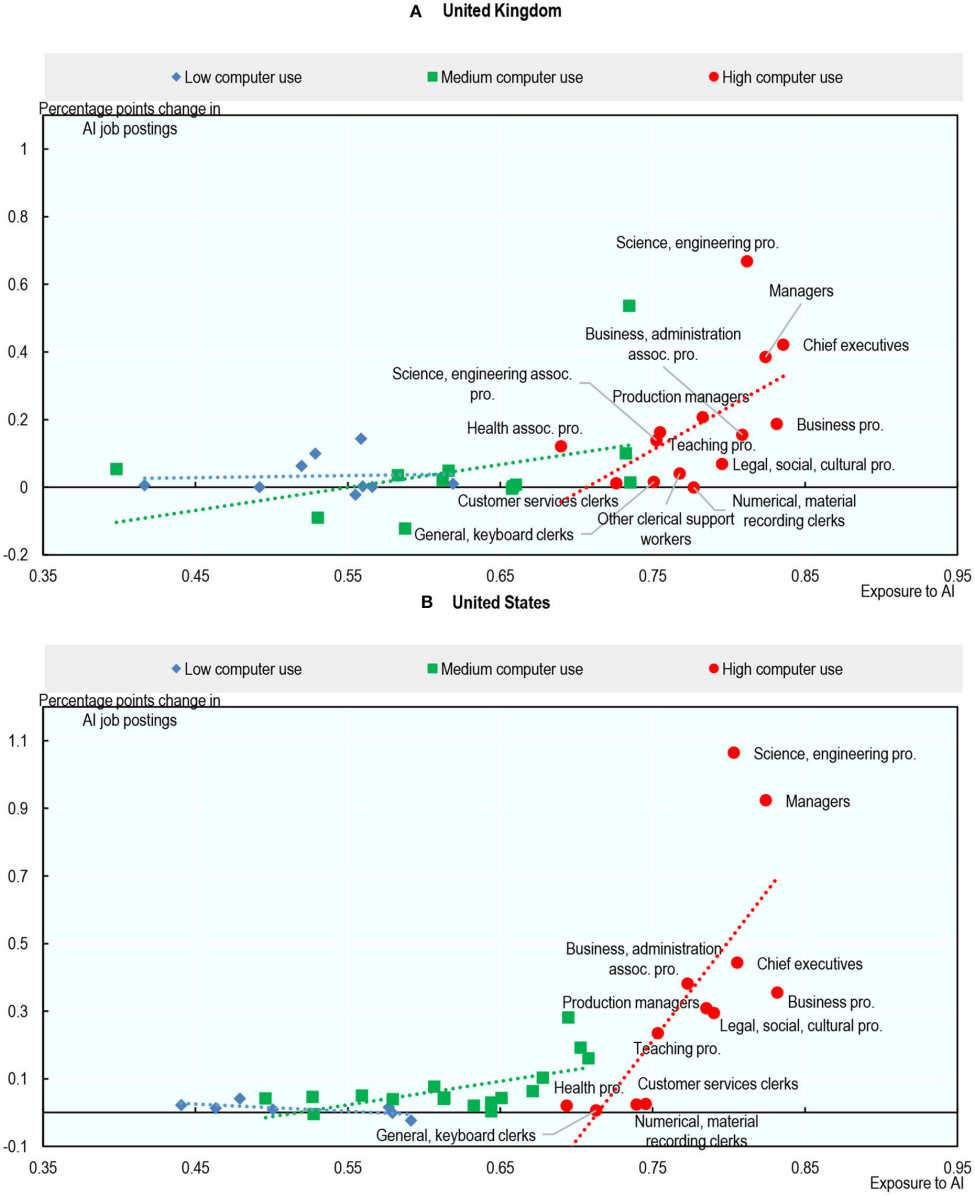
High computer use occupations with higher exposure to AI saw a higher increase in their share of job postings that require AI skills. Percentage point change in the share of job postings that require AI skills (2012–2019) and exposure to AI (2012). The share of job postings that require AI skills in an occupation is taken as a share of the total number of job postings in that occupation. Occupation-country cells are classified into low, medium or high computer use by tercile of computer use applied across the full sample of occupation-country cells. Source: Author' calculations using data from Burning Glass Technologies, PIAAC, and Felten et al. (2019). **(A)** United Kingdom and **(B)** United States.

Indeed, exposure to AI is positively associated with the growth in the demand for AI technical skills, especially in occupations where computer use is high. Figure 15 shows the correlation between the growth in the share of job postings that require AI skills from 2012 to 2019 within occupations and occupation-level exposure to AI for the United Kingdom (Figure 15A) and the United States (Figure 15B), the only countries in the sample with BGT time series available. Occupations are again classified according to their computer use. There is a positive correlation between the growth in the share of job postings requiring AI skills and the AI exposure measure, particularly among occupations where computer use is high. The most exposed of these occupations (Science and Engineering Professionals; Managers; Chief Executives; Business and Administration Professionals; Legal, Social, Cultural professionals) are also experiencing the largest increases in job postings requiring AI skills.

However, the increase in jobs requiring AI skills cannot account for the additional employment growth observed in computer-intensive occupations that are exposed to AI (despite the similarities between the patterns displayed in Figures 13, 15). As highlighted by the different scales in those two charts, the order of magnitude of the correlation between exposure to AI and the percentage change in employment (Figure 13) is more than ten times that of the correlation between exposure to AI and the percentage point change in the share of job postings requiring AI skills (Figure 15)^70^. This is because job postings requiring AI skills remain a very small share of overall job postings. In 2019, on average across the 36 occupations analyzed, job postings that require AI skills accounted for only 0.14% of overall postings in the United Kingdom and 0.24% in the United States. By contrast, across the same 36 occupations, employment grew by 8.82% on average in the United States and 11.15% in the United Kingdom between 2012 and 2019.

## Conclusion

Recent years have seen impressive advances in artificial intelligence (AI) and this has stoked renewed concern about the impact of technological progress on the labor market, including on worker displacement.

This paper looks at the possible links between AI and employment in a cross-country context. It adapts the *AI occupational impact measure* developed by Felten et al. (2018, 2019)—an indicator measuring the degree to which occupations rely on abilities in which AI has made the most progress—and extends it to 23 OECD countries. The indicator, which allows for variations in AI exposure across occupations, as well as within occupations and across countries, is then matched to Labor Force Surveys, to analyse the relationship with employment.

Over the period 2012–2019, employment grew in nearly all occupations analyzed. Overall, there appears to be no clear relationship between AI exposure and employment growth. However, in occupations where computer use is high, greater exposure to AI is linked to higher employment growth. The paper also finds suggestive evidence of a negative relationship between AI exposure and growth in average hours worked among occupations where computer use is low.

While further research is needed to identify the exact mechanisms driving these results, one possible explanation is that partial automation by AI increases productivity directly as well as by shifting the task composition of occupations toward higher value-added tasks. This increase in labor productivity and output counteracts the direct displacement effect of automation through AI for workers with good digital skills, who may find it easier to use AI effectively and shift to non-automatable, higher-value added tasks within their occupations. The opposite could be true for workers with poor digital skills, who may not be able to interact efficiently with AI and thus reap all potential benefits of the technology.

## Data Availability Statement

Publicly available datasets were analyzed in this study. This data can be found at: https://www.oecd.org/skills/piaac/data/.

## Author Contributions

Both authors contributed to the article and approved the submitted version.

## Conflict of Interest

The authors declare that the research was conducted in the absence of any commercial or financial relationships that could be construed as a potential conflict of interest.

## Publisher's Note

All claims expressed in this article are solely those of the authors and do not necessarily represent those of their affiliated organizations, or those of the publisher, the editors and the reviewers. Any product that may be evaluated in this article, or claim that may be made by its manufacturer, is not guaranteed or endorsed by the publisher.

## Footnotes

^2^AI may however be used in robotics (“smart robots”), which blurs the line between the two technologies (Raj and Seamans, 2019). For example, AI has improved the vision of robots, enabling them to identify and sort unorganised objects such as harvested fruit. AI can also be used to transfer knowledge between robots, such as the layout of hospital rooms between cleaning robots (Nolan, 2021).

^3^This can only be the case if an occupation is only partially automated, but depending on the price elasticity of demand for a given product or service, the productivity effect can be strong. For example, during the nineteenth century, 98% of the tasks required to weave fabric were automated, decreasing the price of fabric. Because of highly price elastic demand for fabric, the demand for fabric increased as did the number of weavers (Bessen, 2016).

^4^Education directly increases task-specific human capital as well as the rate of learning-by-doing on the job, at least some of which is task-specific (Gibbons and Waldman, 2004, 2006). This can be seen by looking at the likelihood of lateral moves within the same firm: lateral moves have a direct productivity cost to the firm as workers cannot utilise their entire task-specific human capital stock in another area (e.g., when moving from marketing to logistics). However, accumulating at least some task-specific human capital in a lateral position makes sense if a worker is scheduled to be promoted to a position that oversees both areas. If a worker's task-specific human capital is sufficiently high, however, the immediate productivity loss associated with a lateral move is higher than any expected productivity gain from the lateral move following a promotion. For example, in academic settings, Ph.D., economists are not typically moved to the HR department prior to becoming the dean of a department. Using a large employer-employee linked dataset on executives at US corporations, Jin and Waldman (2019) show that workers with 17 years of education were twice as likely to be laterally moved before promotion than workers with 19 years of education.

^5^An occupation is “exposed” to AI if it has a high intensity in skills that AI can perform, see section What Do These Indicators Measure? for details.

^6^Fossen and Sorgner (2019) use the occupational impact measure developed by Felten et al. (2018, 2019) and the Suitability for Machine Learning indicator developed by Brynjolfsson and Mitchell (2017) and Brynjolfsson et al. (2018) discussed in Section What Do These Indicators Measure?

^7^Acemoglu et al. (2020) use data from Brynjolfsson and Mitchell, 2017; Brynjolfsson et al., 2018, Felten et al. (2018, 2019), and (Webb, 2020) to identify tasks compatible with AI capabilities; and data from online job postings to identify firms that use AI, see Section Indicators of Occupational Exposure to AI for details.

^8^Sectors are available according to the North American Industry classification system (NAICS) for the US and Canada and according to the UK Standard Industrial Classification (SIC) and Singapore Industrial Classification (SSIC) for the UK and Singapore. Occupational codes are available according to the O^*^NET classification for Canada, SOC for the UK, and the US and SSOC for Singapore. These codes can be converted to ISCO at the one-digit level.

^9^This paper uses the same list of skills to look at AI job-postings, see Footnote 44 for the complete list of skills.

^10^To measure importance of skills in job ads, the authors use the Revealed Comparative Advantage (RCA) measure, loaned from trade economics, that weighs the importance of a skill in a job posting up if the number of skills for this specific posting is low, and weighs it down if the skill is ubiquitous in all job adds. That is, the skill “team work” will be generally less important given its ubiquity in all job ads, but its importance in an individual job posting would increase if only few other skills were required for that job.

^11^“Artificial Intelligence,” “Machine Learning,” “Data Science,” “Data Mining,” and “Big Data”.

^12^The indicator is calculated at the division level (19 industries) according to the Australian and New Zealand Standard Industrial Classification Level (ANZSIC).

^13^Abstract strategy games, real-time video games, image recognition, visual question answering, image generation, reading comprehension, language modelling, translation, and speech recognition. Abstract strategy games, for example are defined as “the ability to play abstract games involving sometimes complex strategy and reasoning ability, such as chess, go, or checkers, at a high level.” While the EFF tracks progress on 16 applications, AI has not made any progress on 7 of these over the relevant time period (Felten et al., 2021).

^14^The background of the gig workers is not known and so they may not necessarily be AI experts. This could be a potential weakness of this indicator. In contrast (Tolan et al., 2021) rely on expert assessments for the link between AI applications and worker abilities (Tolan et al., 2021).

^15^At the six digit SOC 2010 occupational level, this can be aggregated across sectors and geographical regions, see Felten et al. (2021).

^16^The abilities are chosen from Hernández-Orallo (2017) to be at an intermediate level of detail, excluding very general abilities that would influence all others, such as general intelligence, and too specific abilities and skills, such as being able to drive a car or music skills. They also exclude any personality traits that do not apply to machines. The abilities are: Memory processing, Sensorimotor interaction, Visual processing, Auditory processing, Attention and search, Planning, sequential decision-making and acting, Comprehension and expression, Communication, Emotion and self-control, Navigation, Conceptualisation, learning and abstraction, Quantitative and logical reasoning, Mind modelling and social interaction, and Metacognition and confidence assessment.

^17^Free and open repository of machine learning code and results, which includes data from several repositories (including EFF, NLPD progress etc.).

^18^An archive kept by the by the Association for the Advancement of Artificial Intelligence (AAI).

^19^AI-related technical skills are identified based on the list provided in Acemoglu et al. (2020), and detailed in Footnote 44.

^20^As with occupations, the industry-level scores are derived using the average frequency with which workers in each industry perform a set of 33 tasks, separately for each country.

^21^The United Kingdom and the United States are the only countries in the sample analysed (see Section Construction of the AI Occupational Exposure Measure) with 2012 Burning Glass Technologies data available, thereby allowing for the examination of trends over the past decade.

^22^The standard deviation of exposure to AI is 0.083 in the United-Kingdom and 0.075 in the United-States. These values are multiplied by the slopes of the linear relationships displayed in Figure 1: 3.90 and 4.95, respectively. The average share of job postings that require AI skills was 0.14% in the United-Kingdom and 0.26% in the United-States in 2012, and this has increased to 0.67 and 0.94%, respectively, in 2019.

^23^The 23 countries are Austria, Belgium, the Czech Republic, Denmark, Estonia, Finland, France, Germany, Greece, Hungary, Ireland, Italy, Lithuania, Mexico, the Netherlands, Norway, Poland, Slovenia, the Slovak Republic, Spain, Sweden, United Kingdom, and the United States.

^24^This paper aims to explore the links between employment and AI deployment in the economy, rather than the direct employment increase due to AI development. Two occupations are particularly likely to be involved in AI development: IT technology professionals and IT technicians. These two occupations both have high levels of exposure to AI and some of the highest employment growth over this paper's observation period, which may be partly related to increased activity in AI development. These occupations may bias the analysis and they are therefore excluded from the sample. Nevertheless, the results are not sensitive to the inclusion of IT technology professionals and IT technicians in the analysis.

^25^A few occupation/country cells are missing due to data unavailability for the construction of the indicator of occupational exposure to AI: Skilled forestry, fishery, hunting workers in Belgium and Germany; Assemblers in Greece; Agricultural, forestry, fishery labourers in Austria and France, and Food preparation assistants in the United Kingdom.

^26^This paper uses BGT data for additional results for the countries for which they are available.

^27^While the three task-based indicators point to the same relationships between exposure to AI and employment, the results are less clearcut for the relationship between exposure to AI and average working hours.

^28^The 33 tasks were then grouped into 12 broad categories to address differences in data availability between types of task. For example, “read letters,” “read bills,” and “write letters” were grouped into one category (“literacy–business”), so that this type of task does not weight more in the final score than tasks types associated with a single PIAAC task (e.g., “dexterity” or “management”). For each ability and each occupation, 12 measures were constructed to reflect the frequency with which workers use the ability in the occupation to perform tasks under the 12 broad task categories. This was done by taking, within each category of tasks, the sum of the frequencies of the tasks assigned to the ability divided by the total number of tasks in the category. Finally, the frequency with which workers use the ability at the two-digit ISCO-08 level and by country was obtained by taking the sum of these 12 measures. The methodology, including the definition of the broad categories of tasks, is adapted from Fernández-Macías and Bisello (2020) and Tolan et al. (2021).

^29^The 17 lost abilities are: control prevision, multilimb coordination, response orientation, reaction time, speed of limb movement, explosive strength, extent flexibility, dynamic flexibility, gross body coordination, gross body equilibrium, far vision, night vision, peripheral vision, glare sensitivity, hearing sensitivity, auditory attention, and sound localization.

^30^Perceptual speed is the ability to quickly and accurately compare similarities and differences among sets of letters, numbers, objects, pictures, or patterns. Speed of closure is the ability to quickly make sense of, combine, and organize information into meaningful patterns. Flexibility of closure is the ability to identify or detect a known pattern (a figure, object, word, or sound) that is hidden in other distracting material.

^31^Only one psychomotor ability has an intermediate score: rate control, which is the ability to time one's movements or the movement of a piece of equipment in anticipation of changes in the speed and/or direction of a moving object or scene.

^32^To get results at the ISCO-08 2-digit level, scores were mapped from the SOC 2010 6-digits classification to the ISCO-08 4-digit classification, and aggregated at the 2-digit level by using average scores weighted by the number of full-time equivalent employees in each occupation in the United States, as provided by Webb (2020) and based on American Community Survey 2010 data.

^33^Averages are unweighted averages across occupations, so that cross-country differences only reflect differences in the ability requirements of occupations between countries, not differences in the occupational composition across countries.

^34^Although specific data on cleaning robots are not available, data from the International Federation of Robotics show that, in 2012, industrial robots were more prevalent in Finland than in Lithuania in all areas for which data are available.

^35^Again, as in the rest of the paper, *exposure to AI* specifically refers to potential automation of tasks, as this is primarily what task-based measures of exposure capture.

^36^On average across countries, there is no clear relationship between AI exposure and gender and age, see Figures A A.4, A A.5 in the Annex.

^37^Employment includes all people engaged in productive activities, whether as employees or self-employed. Employment data is taken from the Mexican National Survey of Occupation and Employment (ENOE), the European Union Labour Force Survey (EU-LFS), and the US Current Population Survey (US-CPS). The occupation classification was mapped to ISCO-08 where necessary. More specifically, the ENOE SINCO occupation code was directly mapped to the ISCO-08 classification. The US-CPS occupation census code variable was first mapped to the SOC 2010 classification. Next, it was mapped to the ISCO-08 classification.

^38^Hours worked refer to the average of individuals' usual weekly hours, which include the number of hours worked during a normal week without any extra-ordinary events (such as leave, public holidays, strikes, sickness, or extra-ordinary overtime).

^39^2012 is available in PIAAC for most countries except Hungary (2017), Lithuania (2014), and Mexico (2017).

^40^Estimated at the average over the sample (37.7 average usual weekly hours).

^41^Mexico is excluded from the analysis of working time due to lack of data.

^42^See Box 1 for more details on Burning Glass Technologies data. The Burning Glass Occupation job classification (derived from SOC 2010) was directly mapped to the ISCO-08 classification.

^43^United Kingdom and the United States are the only countries in the sample with 2012 Burning Glass Technologies data available, thereby allowing for the examination of trends over the past decade.

^44^Job postings that require AI-related technical skills are defined as those that include at least one keyword from the following list: Machine Learning, Computer Vision, Machine Vision, Deep Learning, Virtual Agents, Image Recognition, Natural Language Processing, Speech Recognition, Pattern Recognition, Object Recognition, Neural Networks, AI ChatBot, Supervised Learning, Text Mining, Support Vector Machines, Unsupervised Learning, Image Processing, Mahout, Recommender Systems, Support Vector Machines (SVM), Random Forests, Latent Semantic Analysis, Sentiment Analysis/Opinion Mining, Latent Dirichlet Allocation, Predictive Models, Kernel Methods, Keras, Gradient boosting, OpenCV, Xgboost, Libsvm, Word2Vec, Chatbot, Machine Translation, and Sentiment Classification.

^45^The analysis is performed at the 2-digit level of the International Standard Classification of Occupations 2008 (ISCO-08).

^46^In a second step, *Y*_ij_ will stand for the percentage change in average weekly working hours and the percentage change in the share of part-time workers.

^47^To select software patents, Webb uses an algorithm developed by Bessen and Hunt (2007) which requires one of the keywords “software,” “computer,” or “programme” to be present, but none of the keywords “chip,” “semiconductor,” “bus,” “circuity,” or “circuitry.” To select patents in the field of industrial robots, Webb develops an algorithm that results in the following search criteria: the title and abstract should include “robot” or “manipulate,” and the patent should not fall within the categories: “medical or veterinary science; hygiene” or “physical or chemical processes or apparatus in general”.

^48^They reverse the sign to measure offshorability instead of non-offshorability.

^49^Firpo et al. (2011) define “face-to-face contact” as the average value between the O^*^NET variables “face-to-face discussions,” “establishing and maintaining interpersonal relationships,” “assisting and caring for others,” “performing for or working directly with the public”, and “coaching and developing others.” They define “on-site job” as the average between the O^*^NET variables “inspecting equipment, structures, or material,” “handling and moving objects,” “operating vehicles, mechanized devices, or equipment,” and the mean of “repairing and maintaining mechanical equipment” and “repairing and maintaining electronic equipment”.

^50^All three indices are available by occupation based on U.S. Census occupation codes. They were first mapped to the SOC 2010 6-digits classification and then to the ISCO-08 4-digit classification. They were finally aggregated at the 2-digit level using average scores weighted by the number of full-time equivalent employees in each occupation in the United-States, as provided by Webb (2020) and based on American Community Survey 2010 data.

^51^The tradable sectors considered are agriculture, industry, and financial and insurance activities.

^52^Partial worker substitution in an occupation may increase worker productivity and employment in the same occupation, but also in other occupations and sectors (Autor and Salomons, 2018). These AI-induced productivity effects are relevant to the present cross-occupation analysis to the extent that they predominantly affect the same occupation where AI substitutes for workers. For example, although AI translation algorithms may substitute for part of the work of translators, they may increase the demand for translators by significantly reducing translation costs.

^53^Data are from 2012, with the exception of Hungary (2017), Lithuania (2014), and Mexico (2017).

^54^Low-skill occupations include the ISCO-08 1-digit occupation groups: Services and Sales Workers; and Elementary Occupations. Middle-skill occupations include the groups: Clerical Support Workers; Skilled Agricultural, Forestry, and Fishery Workers; Craft and Related Trades Workers; and Plant and Machine Operators and Assemblers. High-skill occupations include: Managers; Professionals, and Technicians; and Associate Professionals.

^55^In line with Nedelkoska and Quintini (2018), creative tasks include: problem solving—simple problems, and problem solving—complex problems; and social tasks include: teaching, advising, planning for others, communicating, negotiating, influencing, and selling. For each measure, occupation-country cells are then classified into three categories depending on the average frequency with which these tasks are performed (low, medium, and high). These three categories are calculated by applying terciles across the full sample of occupation-country cells. Data are from 2012, with the exception of Hungary (2017), Lithuania (2014), and Mexico (2017).

^56^These results are not displayed but are available on request.

^57^Tables 2, 3 correspond to unweighted regressions, but the results hold when each observation is weighted by the inverse of the number of country observations in the subsample considered, so that each country has the same weight. These results are not displayed but are available on request.

^58^The standard deviation of exposure to AI is 0.067 among high computer use occupations. Multiplying this by the coefficient in Column 4 gives 0.067^*^85.73 = 5.74.

^59^The Webb (2020) indicator is available by occupation based on U.S. Census occupation codes. It was first mapped to the SOC 2010 6-digits classification and then to the ISCO-08 4-digit classification. It was finally aggregated at the 2-digit level by using average scores weighted by the number of full-time equivalent employees in each occupation in the United States, as provided by Webb (2020) and based on American Community Survey 2010 data. The Tolan et al. (2021) indicator is available at the ISCO-08 3-digit level and was aggregated at the 2-digit level by taking average scores.

^60^Although statistically significant on aggregate, the relationships between employment growth and exposure to AI suggested by Table 2 are not visible for some countries.

^61^For productivity-enhancing technologies to have a positive effect on product and labour demand, product demand needs to be price elastic (Bessen, 2019).

^62^The standard deviation of exposure to AI is 0.125 among low computer use occupations. Multiplying this by the coefficient in Column 2 gives 0.125^*^(−4.823) = −0.60.

^63^Estimated at the average working hours among low computer use occupations (37.2 h).

^64^Tables 4, 5 correspond to unweighted regressions, but most of the results hold when each observation is weighted by the inverse of the number of country observations in the subsample considered, so that each country has the same weight. These results are not displayed but are available on request.

^65^Part-time workers are defined as workers usually working 30 hours or less per week in their main job.

^66^As an additional robustness exercise, Table A A.4 in the Appendix replicates the analysis using the score of exposure to AI obtained when using O^*^NET scores of “prevalence” and “importance” of abilities within occupations instead of PIAAC-based measures. The results remain qualitatively unchanged, but the coefficients on exposure to AI are no longer statistically significant on the subsample of occupations where computer use is low, when using working hours as the variable of interest. Tables A A.5, A.6 replicate the analysis using the alternative indicators of exposure to AI constructed by Webb (2020) and Tolan et al. (2021). When using the Webb (2020) indicator, the results hold on the entire sample but are not robust on the subsample of occupations where computer use is low. Using the Tolan et al. (2021) indicator, the results by subgroups hold qualitatively but the coefficients are not statistically significant.

^67^Involuntary part-time workers are defined as part-time workers (i.e., workers working 30 h or less per week) who report either that they could not find a full-time job or that they would like to work more hours.

^68^Although statistically significant on aggregate, the relationships between the percentage change in average usual weekly working hours and exposure to AI suggested by Table 4 are not visible for some countries.

^69^For example, personalised chatbots can partially substitute for travel attendants. Demand forecasting algorithms may facilitate the operation of hotels, including the work of housekeeping supervisors. Travel Attendants and Housekeeping Supervisors both fall into the Personal Service Workers category.

^70^The results of the regression equation (1) on the subsample (of only 26 observations) of high computer use occupations in the United Kingdom and the United States give a coefficient on exposure to AI equal to 151.4 when using percentage employment growth as the variable of interest, which is about forty times greater than the 4.1 obtained when using percentage point change in the share of job postings that require AI skills as the variable of interest.
