# Supplementary material for: Artificial Intelligence and Employment: New Cross-Country Evidence
Source: Front Artif Intell. 2022 May 10;5:832736. doi: 10.3389/frai.2022.832736 (PMC9127971; doi:10.3389/frai.2022.832736)
Supplement: Supplementary file 1 [file Data_Sheet_1.docx]

Additional results

Figure A A.1. Robustness – Alternative measure of exposure to AI based on O*NET data

Note: The average presented is unweighted.

Source: Authors’ calculations using data from the Occupational Information Network (O*NET) database, the Programme for the International Assessment of Adult Competencies (PIAAC) and Felten, Raj and Seamans (2019_[2]_).

Figure A A.2. Robustness – Alternative measure of ability intensity based on O*NET data

Intensity of use of an ability relative to the average across occupations and progress made by AI in relation to that ability, USA

Note: Ability intensity represents the frequency of the use of an ability among Cleaner and helpers (top) or Business professionals (bottom) minus the frequency of the use an that ability averaged across the 36 occupations in the sample.

Source: Authors’ calculations using data from the Occupational Information Network (O*NET) database and Felten, Raj and Seamans (2019_[2]_).

Figure A A.3. The most exposed cleaners and helpers remain half as exposed as the least exposed business professionals

Exposure to AI

Source: Authors’ calculations using data from PIAAC and Felten, Raj and Seamans (2019_[2]_).

Figure A A.4. There is no clear relationship between exposure to AI and gender

Average occupation share (by gender) versus average exposure to AI, across countries and by occupation (2012)

Note: For each gender, occupation shares represent the share of workers of that gender in a particular occupation. Each dot reports the unweighted average across the 23 countries analysed of the share of women or men in an occupation.

Source: Author’ calculations using data from ENOE, EU-LFS, US-CPS, PIAAC and Felten, Raj and Seamans (2019_[2]_).

Figure A A.5. There is no clear relationship between exposure to AI and age

Average occupation share (by age group) versus average exposure to AI, across countries and by occupation (2012)

Note: For each age group, occupation shares represent the share of workers of that group in a particular occupation. Each dot reports the unweighted average across the 23 countries analysed of the share of workers of a particular age group in an occupation.

Source: Author’ calculations using data from ENOE, EU-LFS, US-CPS, PIAAC and Felten, Raj and Seamans (2019_[2]_).

Table A A.1. The average wage level and the prevalence of creative and social tasks matter little in the link between exposure to AI and employment growth

Dependent variable: 2012-2019 % change in employment level

|  | (1) | (2) | (3) | (4) | (5) | (6) | (7) | (8) | (9) |
| --- | --- | --- | --- | --- | --- | --- | --- | --- | --- |
|  | Average wage level | | | Creative tasks | | | Social tasks | | |
|  | Low | Medium | High | Low | Medium | High | Low | Medium | High |
| Exposure to AI | 4.2 | 45.9* | -17.8 | 37.7 | -1.3 | 10.3 | 45.7* | 6.6 | 20.4 |
|  | (21.2) | (26.6) | (45.2) | (25.8) | (33.6) | (50.9) | (26.2) | (23.5) | (43.8) |
|  |  |  |  |  |  |  |  |  |  |
| Share of manufacturing | Yes | Yes | Yes | Yes | Yes | Yes | Yes | Yes | Yes |
| Share of service sector | Yes | Yes | Yes | Yes | Yes | Yes | Yes | Yes | Yes |
| Offshorability | Yes | Yes | Yes | Yes | Yes | Yes | Yes | Yes | Yes |
| Exposure to software | Yes | Yes | Yes | Yes | Yes | Yes | Yes | Yes | Yes |
| Exposure to robots | Yes | Yes | Yes | Yes | Yes | Yes | Yes | Yes | Yes |
| 1-digit occupation FEs | Yes | Yes | Yes | Yes | Yes | Yes | Yes | Yes | Yes |
| Country FEs | Yes | Yes | Yes | Yes | Yes | Yes | Yes | Yes | Yes |
|  |  |  |  |  |  |  |  |  |  |
| Observations | 204 | 319 | 299 | 274 | 274 | 274 | 274 | 274 | 274 |
| R-squared | 0.360 | 0.197 | 0.144 | 0.227 | 0.262 | 0.187 | 0.240 | 0.358 | 0.185 |

Note: Robust standard errors in parentheses. *** p<0.01, ** p<0.05, * p<0.1. Each observation is a country-occupation cell. Column 1-3, 4-6 and 7-9 show the results of regression equation (1) applied to the subsamples obtained by splitting the overall sample by average wage level, prevalence of creative tasks and prevalence of social tasks respectively. The average wage level corresponds to the country-invariant wage-based classification used in (Goos, Manning and Salomons, 2014_[28]_). Occupation-country cells are classified into low, medium or high prevalence of creative tasks/social tasks by tercile of prevalence of creative tasks/social tasks applied across the full sample of occupation-country cells. All columns include the full set of controls used in Table 3 Column 5. Offshorability is an occupation-level measure from Autor and Dorn (2013_[24]_) based on data from the United States. Exposure to software and exposure to robots are occupation-level measures developed by Webb (2020_[7]_) based on data from the United States. The share of tradable sector represents the 2012 share of workers in the country-occupation cell working in: agriculture, industry, and financial and insurance activities.

Source: Authors’ calculations using data from ENOE, EU-LFS, US-CPS, PIAAC, Autor and Dorn (2013_[24]_), Felten, Raj and Seamans (2019_[2]_) and Webb (2020_[7]_).

Table A A.2. Exposure to AI and employment growth – O*NET

Dependent variable: 2012-2019 % change in employment level

|  | (1)  All occupations | (2)  Low computer use | (3)  Medium computer use | (4)  High computer use |
| --- | --- | --- | --- | --- |
| Exposure to AI (O*NET) | 17.9*** | 9.1 | -5.0 | 58.8** |
|  | (6.3) | (22.5) | (14.4) | (23.7) |
|  |  |  |  |  |
| Country FEs | Yes | Yes | Yes | Yes |
| Observations | 822 | 274 | 274 | 274 |
| R-squared | 0.063 | 0.128 | 0.172 | 0.094 |

Note: Robust standard errors in parentheses. *** p<0.01, ** p<0.05, * p<0.1. Each observation is a country-occupation cell. Each column shows the results of regression equation (1) applied to one of the subsamples obtained by splitting the overall sample by level of computer use. Exposure to AI is based on O*NET scores of “prevalence” and “importance” of abilities within occupations instead of PIAAC data. Occupation-country cells are classified into low, medium or high computer use by tercile of computer use applied across the full sample of occupation-country cells.

Source: Authors’ calculations using data from ENOE, EU-LFS, US-CPS, PIAAC, O*NET and Felten, Raj and Seamans (2019_[2]_).

Table A A.3. Exposure to AI and employment growth – Alternative measures of exposure to AI

Dependent variable: 2012-2019 % change in employment level

|  | (1)  All occupations | (2)  Low computer use | (3)  Medium computer use | (4)  High computer use | (5)  All occupations | (6)  Low computer use | (7)  Medium computer use | (8)  High computer use |
| --- | --- | --- | --- | --- | --- | --- | --- | --- |
| Exposure to AI (Webb) | -0.0364 | -0.479*** | -0.0304 | 0.283*** |  |  |  |  |
|  | (0.0544) | (0.0912) | (0.0679) | (0.104) |  |  |  |  |
| Exposure to AI (Tolan) |  |  |  |  | 1.9 | -37.5*** | -12.4** | 15.9 |
|  |  |  |  |  | (4.0) | (9.8) | (5.8) | (12.0) |
|  |  |  |  |  |  |  |  |  |
| Observations | 822 | 274 | 274 | 274 | 822 | 274 | 274 | 274 |
| R-squared | 0.053 | 0.214 | 0.172 | 0.099 | 0.052 | 0.171 | 0.183 | 0.083 |

Note: Robust standard errors in parentheses. *** p<0.01, ** p<0.05, * p<0.1. Each observation is a country-occupation cell. Each column shows the results of regression equation (1) applied to one of the subsamples obtained by splitting the overall sample by level of computer use. The dependent variable is the percentage change in employment levels. Exposure to AI is based on the indicators constructed by Webb (2020_[7]_) and Tolan et al. (2021_[18]_), and described in Section 2.3. Occupation-country cells are classified into low, medium or high computer use by tercile of computer use applied across the full sample of occupation-country cells.

Source: Authors’ calculations using data from ENOE, EU-LFS, US-CPS, Webb (2020_[7]_) and Tolan et al. (2021_[18]_).

Table A A.4. Exposure to AI and working time – O*NET

|  | (1) | (2) | (3) | (4) | (5) | (6) | (7) | (8) |
| --- | --- | --- | --- | --- | --- | --- | --- | --- |
|  | Dependent variable: 2012-2019 % change in working hours | | | | Dependent variable: 2012-2019 % change in part-time employment | | | |
|  | All occupations | Low computer use | Medium computer use | High computer use | All occupations | Low computer use | Medium computer use | High computer use |
| Exposure to AI (O*NET) | -2.4*** | -4.4 | -3.0 | -2.1 | 10.2 | 74.7** | -1.7 | -15.3 |
|  | (0.9) | (3.1) | (2.5) | (2.5) | (10.1) | (36.8) | (62.6) | (46.4) |
|  |  |  |  |  |  |  |  |  |
| Country FEs | Yes | Yes | Yes | Yes | Yes | Yes | Yes | Yes |
| Observations | 781 | 252 | 261 | 268 | 781 | 252 | 261 | 268 |
| R-squared | 0.141 | 0.128 | 0.208 | 0.303 | 0.142 | 0.207 | 0.192 | 0.212 |

Note: Robust standard errors in parentheses. *** p<0.01, ** p<0.05, * p<0.1. Each observation is a country-occupation cell. Each column shows the results of regression equation (1) applied to one of the subsamples obtained by splitting the overall sample by level of computer use. In columns 1-4, the dependent variable is the percentage change in average usual weekly working hours. In columns 5-8, the dependent variable is the percentage change in the share of part-time workers. Exposure to AI is based on O*NET scores of “prevalence” and “importance” of abilities within occupations instead of PIAAC data. Occupation-country cells are classified into low, medium or high computer use by tercile of computer use applied across the full sample of occupation-country cells. Mexico is excluded from the analysis of working time due to data availability.

Source: Authors’ calculations using data from EU-LFS, US-CPS, PIAAC, O*NET and Felten, Raj and Seamans (2019_[2]_).

Table A A.5. Exposure to AI and average working hours – Alternative measures of exposure to AI

Dependent variable: 2012-2019 % change in working hours

|  | (1)  All occupations | (2)  Low computer use | (3)  Medium computer use | (4)  High computer use | (5)  All occupations | (6)  Low computer use | (7)  Medium computer use | (8)  High computer use |
| --- | --- | --- | --- | --- | --- | --- | --- | --- |
| Exposure to AI (Webb) | -0.0212*** | -0.00423 | -0.0290** | -0.0226** |  |  |  |  |
|  | (0.00702) | (0.0170) | (0.0121) | (0.00883) |  |  |  |  |
| Exposure to AI (Tolan) |  |  |  |  | -0.466 | -1.849 | 2.161 | 1.858 |
|  |  |  |  |  | (0.638) | (1.701) | (1.320) | (1.168) |
|  |  |  |  |  |  |  |  |  |
| Observations | 786 | 254 | 262 | 270 | 786 | 254 | 262 | 270 |
| R-squared | 0.140 | 0.123 | 0.224 | 0.314 | 0.132 | 0.126 | 0.213 | 0.310 |

Note: Robust standard errors in parentheses. *** p<0.01, ** p<0.05, * p<0.1. Each observation is a country-occupation cell. The dependent variable is the percentage change in average usual weekly working hours. Exposure to AI is based on the indicators constructed by Webb (2020_[7]_) and Tolan et al. (2021_[18]_), and described in Section 2.3. Each column shows the results of regression equation (1) applied to one of the subsamples obtained by splitting the overall sample by level of computer use. Occupation-country cells are classified into low, medium or high computer use by tercile of computer use applied across the full sample of occupation-country cells. Mexico is excluded from the analysis of working time due to data availability.

Source: Authors’ calculations using data from EU-LFS, US-CPS, PIAAC, Webb (2020_[7]_) and Tolan et al. (2021_[18]_).

Table A A.6. Exposure to AI and part-time employment – Alternative measures of exposure to AI

Dependent variable: 2012-2019 % change in part-time employment

|  | (1)  All occupations | (2)  Low computer use | (3)  Medium computer use | (4)  High computer use | (5)  All occupations | (6)  Low computer use | (7)  Medium computer use | (8)  High computer use |
| --- | --- | --- | --- | --- | --- | --- | --- | --- |
| Exposure to AI (Webb) | 0.278** | 0.110 | 0.591** | 0.0606 |  |  |  |  |
|  | (0.118) | (0.148) | (0.275) | (0.156) |  |  |  |  |
| Exposure to AI (Tolan) |  |  |  |  | -3.2 | 36.6 | -32.2 | -20.1 |
|  |  |  |  |  | (6.5) | (24.7) | (20.3) | (15.8) |
|  |  |  |  |  |  |  |  |  |
| Observations | 781 | 252 | 261 | 268 | 781 | 252 | 261 | 268 |
| R-squared | 0.148 | 0.195 | 0.209 | 0.211 | 0.142 | 0.206 | 0.197 | 0.216 |

Note: Robust standard errors in parentheses. *** p<0.01, ** p<0.05, * p<0.1. Each observation is a country-occupation cell. The dependent variable is the percentage change in the occupation-level share of part-time workers. Exposure to AI is based on the indicators constructed by Webb (2020_[7]_) and Tolan et al. (2021_[18]_), and described in Section 2.3. Each column shows the results of regression equation (1) applied to one of the subsamples obtained by splitting the overall sample by level of computer use. Occupation-country cells are classified into low, medium or high computer use by tercile of computer use applied across the full sample of occupation-country cells. Mexico is excluded from the analysis of working time due to data availability.

Source: Authors’ calculations using data from EU-LFS, US-CPS, PIAAC, Webb (2020_[7]_) and Tolan et al. (2021_[18]_).

Table A A.7. Exposure to AI is positively associated with growth in involuntary part-time employment in occupations where computer use is low

Dependent variable: 2012-2019 percentage point change in involuntary part-time employment

|  | (1)  All occupations | (2)  Low computer use | (3)  Medium computer use | (4)  High computer use |
| --- | --- | --- | --- | --- |
| Exposure to AI | 3.6*** | 9.0*** | 6.4*** | 0.5 |
|  | (0.8) | (1.8) | (2.1) | (1.8) |
|  |  |  |  |  |
| Observations | 781 | 252 | 261 | 268 |
| R-squared | 0.389 | 0.466 | 0.400 | 0.633 |

Note: Robust standard errors in parentheses. *** p<0.01, ** p<0.05, * p<0.1. Each observation is a country-occupation cell. The dependent variable is the percentage point change in the occupation-level share of involuntary part-time workers. Percentage point change is preferred over percentage change because the share of involuntary part-time workers is equal to zero in some occupations in 2012. Each column shows the results of regression equation (1) applied to one of the subsamples obtained by splitting the overall sample by level of computer use. Occupation-country cells are classified into low, medium or high computer use by tercile of computer use applied across the full sample of occupation-country cells. Mexico is excluded from the analysis of working time due to data availability.

Source: Authors’ calculations using data from EU-LFS, US-CPS, PIAAC and Felten, Raj and Seamans (2019_[2]_).
